# Emergent Self-Organized Criticality in Gene Expression Dynamics: Temporal Development of Global Phase Transition Revealed in a Cancer Cell Line

**DOI:** 10.1371/journal.pone.0128565

**Published:** 2015-06-11

**Authors:** Masa Tsuchiya, Alessandro Giuliani, Midori Hashimoto, Jekaterina Erenpreisa, Kenichi Yoshikawa

**Affiliations:** 1 Systems Biology Program, School of Media and Governance, Keio University, Fujisawa, Japan; 2 Environment and Health Department, Istituto Superiore di Sanitá, Rome, Italy; 3 Graduate School of Frontier Science, The University of Tokyo, Kashiwa, Japan; 4 Latvian Biomedical Research & Study Centre, Riga, Latvia; 5 Faculty of Life and Medical Sciences, Doshisha University, Kyotanabe, Japan; University of Zurich, SWITZERLAND

## Abstract

**Background:**

The underlying mechanism of dynamic control of the genome-wide expression is a fundamental issue in bioscience. We addressed it in terms of phase transition by a systemic approach based on both density analysis and characteristics of temporal fluctuation for the time-course mRNA expression in differentiating MCF-7 breast cancer cells.

**Methodology:**

In a recent work, we suggested criticality as an essential aspect of dynamic control of genome-wide gene expression. Criticality was evident by a unimodal-bimodal transition through flattened unimodal expression profile. The flatness on the transition suggests the existence of a critical transition at which up- and down-regulated expression is balanced. Mean field (averaging) behavior of mRNAs based on the temporal expression changes reveals a sandpile type of transition in the flattened profile. Furthermore, around the transition, a self-similar unimodal-bimodal transition of the whole expression occurs in the density profile of an ensemble of mRNA expression. These singular and scaling behaviors identify the transition as the expression phase transition driven by self-organized criticality (SOC).

**Principal Findings:**

Emergent properties of SOC through a mean field approach are revealed: i) SOC, as a form of genomic phase transition, consolidates distinct critical states of expression, ii) Coupling of coherent stochastic oscillations between critical states on different time-scales gives rise to SOC, and iii) Specific gene clusters (barcode genes) ranging in size from kbp to Mbp reveal similar SOC to genome-wide mRNA expression and ON-OFF synchronization to critical states. This suggests that the cooperative gene regulation of topological genome sub-units is mediated by the coherent phase transitions of megadomain-scaled conformations between compact and swollen chromatin states.

**Conclusion and Significance:**

In summary, our study provides not only a systemic method to demonstrate SOC in whole-genome expression, but also introduces novel, physically grounded concepts for a breakthrough in the study of biological regulation.

## Introduction

Inside living cells, a large number of molecular species (DNA, RNA, proteins, and metabolites) interact with each other in response to environmental stimuli. It is intriguing to consider how cells can select specific pathways, such as differentiation or immune response, out of the vast number of combinatorial possibilities arising from complex multi-molecular interactions. This robust organization goes hand-in-hand with an extreme sensitivity to specific stimuli: e.g., in mammalian stem cells, a few key transcription factors, such as Oct4, Sox2, and Nanog or Yamanaka’s factors in iPS cells, coordinate the expression of thousands of genes [1–3].

The coordinated control of the expression of a large number of genes in a cell must overcome several difficulties. The presence of stochastic noise due to the intrinsic effect of a low copy number of specific gene mRNAs per cell and the lack of a sufficient number of molecules to reach a thermodynamic limit, may lead to the following problems, respectively:

1. The relative abundance of genetic products, if based solely on a very large number of specific key-lock interactions without systemic contributions from the molecular micro-environment, is expected to undergo wild variations and substantial instability [4], and
2. By considering the thermal and number fluctuations on interactions involving extremely small integer numbers of key and lock molecules in nucleus, the central limit theorem should break down [5], which suggests that kinetic differential equation approaches adapting the parameters of continuous variables are invalid.

Thus, it is natural to abandon a ‘single molecule’ level of explanation when considering self-organization into discrete ‘phenotypic states’ as stable attractor states in the gene-expression landscape [6–8].

The concept of attractor envisages the system as evolving toward a preferred (minimal energy) state called an attractor set, which is formalized as a point, a curve, or a manifold in the state space spanned by the relative concentrations of a huge number of molecular players. The emergence of a favored ‘globally convergent’ solution that attracts the system dynamics overcomes the problem of stochastic fluctuations related to a gene-by-gene regulation paradigm. This can happen in the presence of a general ‘energy field’ that shapes a rugged landscape where the valleys correspond to attractor states. The shape of the ‘energy field’ would be discussed in terms of the symmetry argument of Landau [9].

To interpret biological regulation within the framework of physics (even if still largely phenomenological), we must eliminate the need for Maxwell’s demons [10], i.e., intelligent agents that actively drive the system toward a desired goal. The life sciences literature offers many of such agents: e.g., proteins that ‘see’ or ‘recruit’ other proteins that care for each pathway, impeding superposition through simultaneous regulation (the same need is clearly set forth by Laue and Demeler [11]). An attractor-based global dynamics under thermodynamically open conditions for all living matter enables regulation without the need for such intelligent agents. Then, seeing a cell dynamically controlling genome-wide expression, a fundamental question for such genomic activity arises:

*What is the ‘driving force’ that attracts the entire system toward a few preferred global states, thus making the genome act as a single integrated system?*

Statistical mechanics postulates that energetically preferred configurations of a system arise through the satisfaction of relationships among its constituent parts subjected to external constraints. These correlations shape the state space of the cell as an ‘epigenetic landscape’. In Waddington’s original formulation [12,13], an epigenetic landscape is the set of ‘causal interactions between genes and their products, which bring the phenotype into being’ [13].

Similar to the standard framework of classical thermodynamics, an epigenetic landscape can be interpreted as a free energy profile based on the entire ensemble of simultaneous interactions [14], with the free energy of each molecule expressed as Δ*G* = *n*Δ*H* − *T*Δ*S* (n = number of binding sites). Since Tompa and Rose estimated the presence of a transfinite number of simultaneous interactions, in the order of 10^7200^ for a simple organism such as yeast [15], it would be impossible to evaluate such a free energy profile.

From physical chemistry, we know that a collection of molecules can pass from a gas to a liquid and solid phase according to the temperature. In state changes such as the phase transition that occurs in a ferromagnetic material at the Curie temperature (*T*_c_), the spins of different molecules behave as a single coherent object to show spontaneous magnetization below *T*_c_, whereas above *T*_c_, the thermodynamic motion of molecules destroys the ordering of spins. Furthermore, in a nonlinear environment, spontaneous symmetry breaking is possible in the case of a single-well to double-well free energy transition accompanied by the bifurcation of new attractor states (as energy local minima). Through symmetry breaking, multi-stable attractor states emerge spontaneously; the possibility of a rich attractor landscape (Hopfield model) was demonstrated in the case of frustrated systems [16]. The Hopfield model depicts the system as being embedded in a non-uniform state space (an ensemble of all possible system configurations) characterized by a so-called ‘rugged landscape’ in which the energy minima (valleys of the landscape, quasi-equilibrium configurations) correspond to attractor states. Each system accommodates to the nearest energy minimum, consistent with the marked ‘context dependence’ (e.g., sensitivity to the microenvironment) of biological regulation.

Phase transitions show how the choice of ‘global modes’ can be finely tuned by a few control parameters (such as temperature) that determine the general fate of the system. As postulated by Yamanaka [17], the reprogramming of cell states can be achieved only very rarely because of the presence of very high kinetic barriers. Nevertheless, the fact that such reprogramming can occur means that the corresponding states are ‘allowed’. In other words, only the relative probability of these states (and not their existence *per se*) depends upon environmental conditions eliciting a ‘preferred state’ out of many possible configurations.

**Fig 1:**
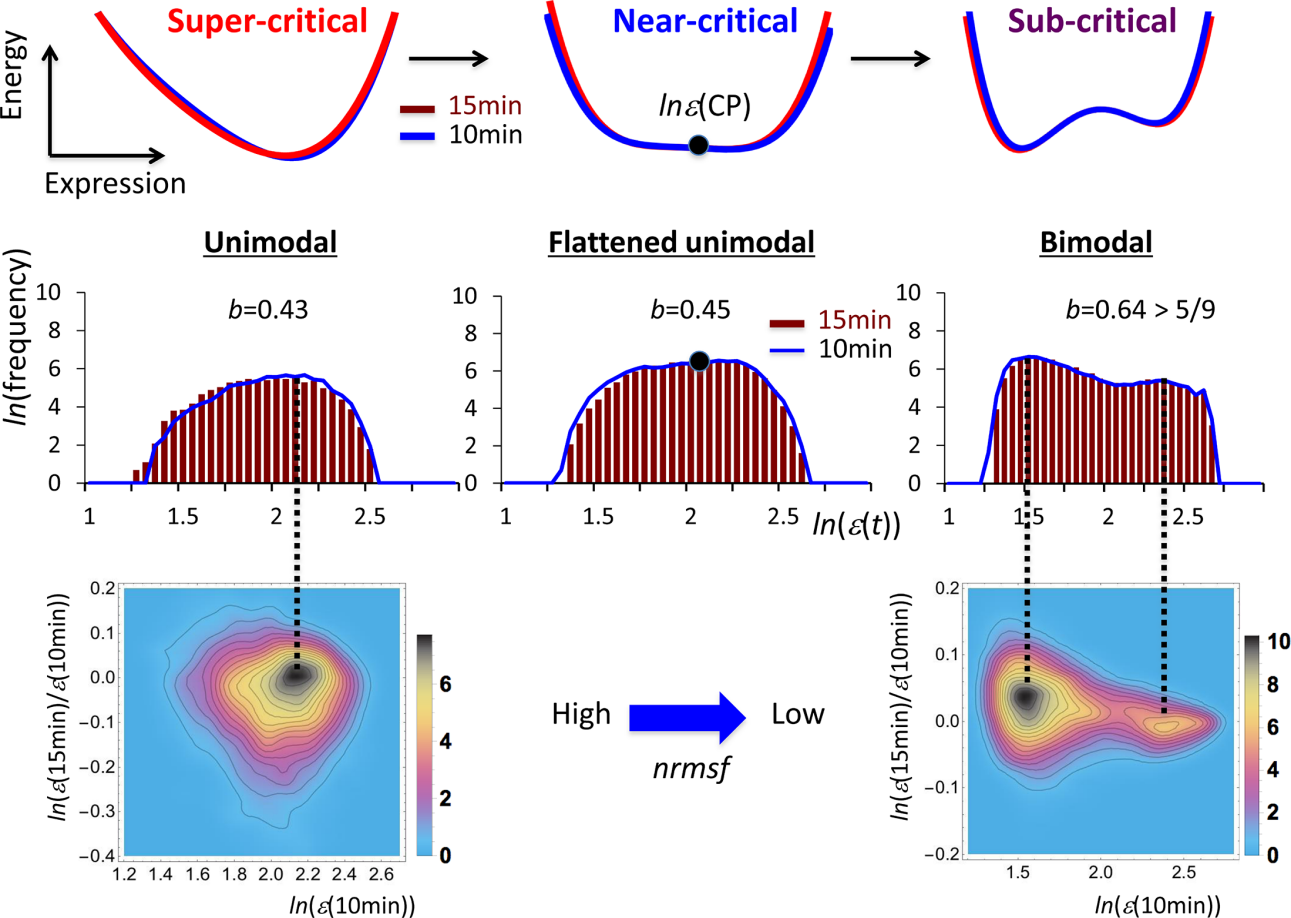
Criticality mirrored by the unimodal-bimodal transition through the flattened unimodality. Criticality of the whole expression at 10–15 min of MCF-7 cell stimulated by HRG exhibits three distinct response domains going from higher to lower *nrmsf* (left to right in the figure): (left) dynamic domain (*nrmsf*> 0.16; unimodal profile: *N* = 3269 mRNAs), (middle) transit domain (0.08 <*nrmsf*< 0.16 flattened unimodal profile: *N* = 9707 mRNAs), (right) static domain (*nrmsf* < 0.21; bimodal profile: *N* = 9059 mRNAs). First row shows the corresponding putative energy profiles (*x*-axis: states; *y*-axis: energy, here specified in abstract terms referring to a physical system undergoing a transition) from a single-well to double-well profiles through flattened single-well profile (blue: 10 min; 15 min: red). These energy profiles should correspond to free energy in terms of the symmetry argument of Landau. Second row shows frequency distributions of mRNA expression from unimodal to bimodal distribution through a flattened unimodal distribution (*b*: Sarle's bimodality coefficient; *x*: natural log of expression, *ln*(*ε*(t)) and y: natural log of frequency; blue polygonal line: 10 min; red histogram: 15 min); Third row reports the density profile in the regulatory space (*x*: natural log of expression, *ln*(*ε*(10min)) at 10 min vs. *y*: log of the change in expression at 10–15 min, *ln*(*ε*(15min)/*ε*(10min))) showing clear unimodal to bimodal transition (color bars: probability density). Match of peaks of the histograms and density profiles confirms the statistical reliability of the unimodal-bimodal transition of frequency distribution. The temporal invariant flatness of energy profile suggests the existence of the critical point (CP) (*ln*(*ε*(CP)), black solid circle), which is the point where up- and down-regulation balance, i.e., the point where the change in expression between different time points is around zero.

We have recently suggested the presence of criticality regarding whole mRNA expression on the model of an early response to growth factors in a MCF-7 breast cancer cell population [18]. Criticality characterizes distinct expression domains: dynamic, transit and static domains according to the degree of temporal variation in expression (*nrmsf*: Materials and Methods). Fig 1 shows a unimodal-bimodal transition through flattened unimodal expression profile. Moreover, the temporal development of criticality (dynamic criticality) gives rise to an autonomous bistable switch (ABS) for each domain with a pendulum oscillatory system of coherent expression states (CESs) [18].

Here we get a deeper insight into dynamic criticality by the demonstration of the existence of a critical transition, where a global phase transition in the whole gene expression profile takes place. Around the transition, a clear shift in the frequency profiles of the ensemble of (thousands) stochastic mRNA expression occurs, from unimodal to bimodal, through flattening of the unimodal profile. The result clearly indicates that the dynamics of gene expression show some peculiar (scaling and singular) features of critical behavior near a transition of self-organized criticality (SOC). Then we conducted a correlation analysis of expression groups sorted according to normalized root-mean-square-fluctuation (*nrmsf*: refer to Materials and Methods) to demonstrate temporal development of global phase transition and to elucidate an underlying mechanism for the formation of SOC relative to an early response (the first 30 min) to growth factors in a MCF-7 breast cancer cell population.

**Fig 2:**
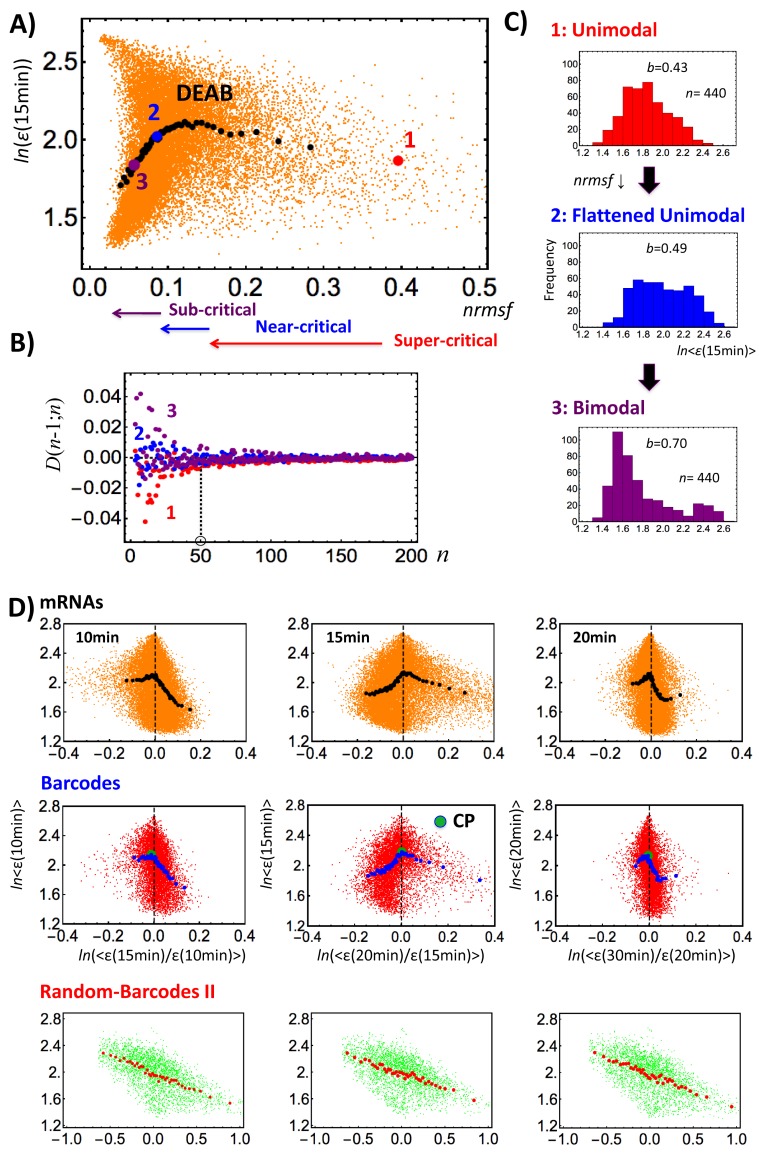
Mean field behavior of the whole mRNA expression and sandplie type singular behaviors. Dynamic emergent averaging behavior (DEAB) of the expression (mean field behavior) reveals a unimodal to bimodal transition through a flattened unimodality: A) Scattered single mRNA expression (orange dot) overlays with DEAB of the expression (black solid dot) for the HRG response of MCF-7 cells at 15 min on a space spanned by *ln*(*ε*(15min)) and ln(1-*nrmsf*) with the region of *nrmsf* for three critical states. DEAB of the expression presents ensemble of points, {<*nrmsf*>, *ln*<*ε*(15min)>} (group size: *n* = 440 mRNAs). B) The difference of points between neighboring group sizes: *D*(*n*;*n*-1) = {(*x*_n_−*x*_n-1_) + (*y*_n_−*y*_n-1_)} converges to zero for three points (1: red, 2: blue, 3: purple) on DEAB (*n*> 50), which depicts the law of large numbers in statistics in that average value converges into a certain value as the ensemble size, *n* is increased. The *x*-axis represents group size, *n* and the *y*-axis represents D(*n*;*n*-1). An initial element of a group (*n* = 1) builds from its highest *nrmsf*. C) Frequency (histogram with bin = 0.1) distribution of three group points (1, 2, 3) on DEAB reveals a unimodal (1: *b* = 0.43) to bimodal (3: *b* = 0.70 >5/9) transition through a flattened unimodality (2: *b* = 0.49), where *b* is Sarle's bimodality coefficient for a finite sample when b> 5/9 may indicate a bimodal or multimodal distribution. The result shows that a transition point exists at a flattened profile. Sandpile type singular behaviors are revealed from grouping by expression change: D) The grouping of mRNA expression (different mean-field from one based on *nrmsf*) at *t* = *t*_*j*_ according to the degree of expression change at *t*_*j+1*_ *–t*_*j*_ (*j* = 10, 15, 20, 30 min) reveals a sharp transition similar to the sandpile model—top row for mRNAs (group size: *n* = 440), and middle row for barcode genes (*n* = 182; refer to Fig 8) overlaying with single expression distribution (orange: mRNA; red: barcode). On the contrary, randomized barcode genes (*n* = 78; random barcode II; see the main text) show no evidence of transition (bottom row; green: single barcode) in the expression vs. expression change plane. Left panels: 10 min vs. 10–15 min; Middle panels: 15 min. vs. 15–20 min.; Right panels: 20 min. vs. 20–30 min, <> represents simple arithmetic mean over an ensemble or a group.

Here, it is important to stress that a single gene level is not the correct scale where to address the real emergent nature of global genome response through SOC. As shown in Fig 2A, single expression just shows a scattered stochastic expression distribution. Therefore, a second question emerges:

How can the occurrence of a global phase transition through criticality be affirmed in such stochastic expression?

The basic aim of our report is to show the occurrence of self-organized criticality (SOC) in the whole expression through a mean field approach, where at the single gene level, expression is stochastic, fluctuating around the average expression value of each group along the global profile. Fig 2 shows the existence of a smooth curve (manifold; Fig 2A) arisen by grouping mRNAs, which suggests the existence of a mean field behavior (group size: *n*> 50; Fig 2B) in the genome-wide expression dynamics. Mean field behavior implies the presence of simple governing principles in physical many-body (e.g., molecular) systems such as spontaneous symmetry breaking in critical phenomena [19].

SOC is an emergent property exhibited in a mean field (averaging) behavior; thus, the grouping together with the determination of minimal group size (threshold) for characteristic behaviors of SOC will be explored.

The choice of *nrmsf* for ordering gene expressions stems from the consolidated notion that entity of gene expression scales with the fractal aggregation state of the chromatin; *nrmsf* should be related to the physical plasticity of genomic DNA, i.e., a higher *nrmsf* should be associated with a more pliable DNA structure, especially in its higher-order structure. Hence, *nrmsf* (i.e., the spatial/temporal variance of elements) should correspond to the degree of fluctuation/freedom in statistical thermodynamics. To highlight the biophysical role of the observed behavior on *nrmsf*, we will elucidate quantitative relationship of ensemble average between *nrmsf* and mRNA expression through their power law behavior exhibited in SOC.

Finally, the link between chromatin aggregation and gene expression is a too coarse grain concept; thus, it is crucially important to look for the biophysical origin of self-organized criticality. In this study we looked for suitable observables associated with coordinating transitional behaviors at the chromosome level, which would support the hypothesis that the structural transition of the chromatin is the biophysical proximate cause of genome-wide regulation. Therefore, these findings together with recent advances in full-genome sequencing and chromatin capture techniques are expected to open new horizons on epigenomics as well as cell biology.

## Results

### Emergent Self-Organized Criticality Through Mean Field Consolidates Critical States of Expression

We grouped the entire mRNA expression profile of MCF-7 cells into *m* equally populated groups at *t* = *t*_*j*_ (*j* = 1, 2,.., 17) in terms of increasing *nrmsf* (see Materials and Methods). This grouping showed characteristic time-dependent correlations among the average values of groups with all-or-none responses to heregulin (HRG) and epidermal growth factor (EGF) (biphasic statistics) at around 10–20 min. The emergent collective behavior relative to the ensemble of genes for both mRNA expression as such and temporal changes in expression suggest criticality [18].

Fig 1 shows that the ensemble of the whole mRNA expression according to *nrmsf* (expression variance) exhibits three critical states (see below) showing a unimodal-bimodal transition through flattened unimodal profile of mRNA expression (details in [18]), and that the flattened profile is almost temporally invariant at 15–20 min. Interestingly, even a smaller ensemble from each critical state reveals a unimodal- flattened unimodal- bimodal transition (Fig 2C), suggesting the existence of a scaling behavior in criticality.

The scenario of transition between different symmetries found on the *nrmsf* suggests that a phase transition is expected to occur through a temporally invariant flattened energy profile; thus critical point (CP) of the transition should exist around a point at which up- and down-regulated expression is balanced, i.e., the change in expression (expression change) between different time points is zero. Note here that the critical point in a mean field indicates a critical transition, which drives an ensemble of thousands of expressions.

Therefore, as the next, we take another mean field approach, the grouping of mRNA expression at *t* = *t*_*j*_ according to the degree of expression change at *t*_j+1_ –*t*_*j*_ (*t*_*j*_ = 0, 10, 15, 20, 30 min,…). In the plane of expression change versus expression, Fig 2D shows a sharp transition similar to the sandpile model [20,21] as a mean field behavior, where the singular point exists near zero expression change. It is worth reminding that a sandpile is the first and most common model of self-organized-criticality.

This singular behavior is also present in the space of *nrmsf* versus expression (data not shown), which confirms CP existence. Hence, CP occurs around zero expression change as expected, and the position of CP in terms of expression and *nrmsf* is determined. CP is around the boundary between low and high-expression (*ln*(*ε*) = 2.075; see the definition of expression level in [18])—it is the region of the balance between up- and down-regulations, and *nrmsf* value of CP is almost temporal invariant (*nrmsf* ~0.09, around the middle place from the highest).

**Fig 3:**
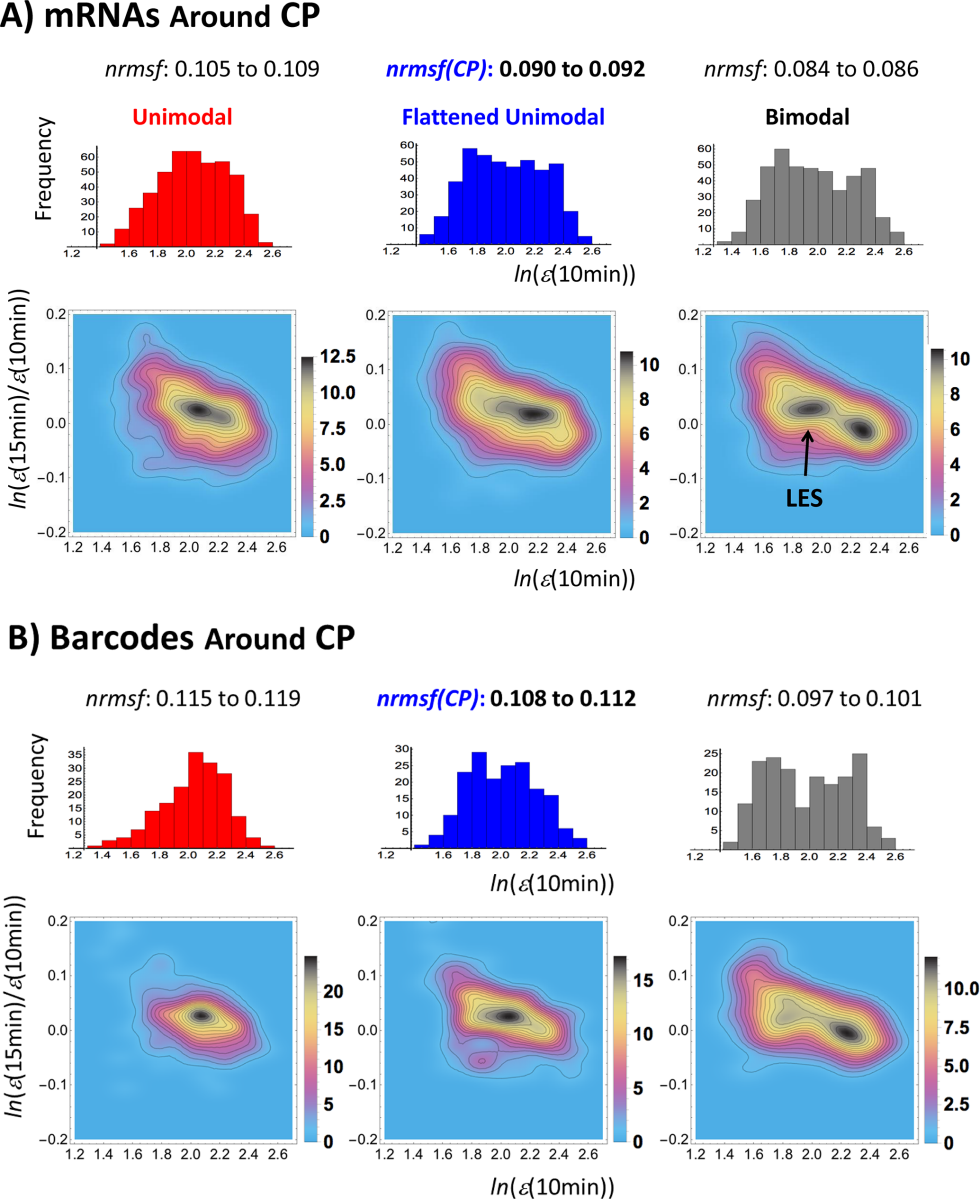
Self-similar power law behavior around CP of mRNA expression and barcode genes. Panel A): First row- frequency distribution (bin size = 0.1) of mRNAs (*n* = 440 mRNAs) at 10 min shows a unimodal to bimodal change around critical point (0.090 <*nrmsf*< 0.092). The *x*-axis represents the natural log of mRNA expression, *ln*(*ε*(10min)) for a specific range of *nrmsf*: unimodal (left panel: 0.105 <*nrmsf*< 0.109), flattened unimodal (middle panel: 0.090 <*nrmsf*< 0.092), and bimodal (right panel: 0.084 <*nrmsf*< 0.086). The y-axis represents frequency of expression. Second row- the corresponding probability density profile in the regulatory space—expression vs. expression change in log scale with probability density (color bars) confirms the unimodal to bimodal transition through flattened unimodality, where a black arrow points to the bifurcation of low-expression state (LES). Panel B): First row—the frequency distribution of barcode genes (*n* = 182 barcodes) shows a unimodal to bimodal change around a critical point (0.108 <*nrmsf*< 0.112) for unimodal (left), flattened (middle) and bimodal (right) distributions. Second row—this is confirmed by probability density profile in the regulatory space. Both mRNAs and barcode genes on chromosomes reveal the existence of self-similar power law (scaling) behavior around CP analogous to that of the whole mRNA expression (see Fig 1), which is an essential characteristic of SOC.

Next, we investigate expression behavior around CP. Shu and colleagues [22] demonstrated, by means of density analysis of noisy gene-expression profiles, the robustness of gene expression clustering. Thus, we applied density analysis to show a hill like probability density function in expression space (see examples in Figs 3 and 7 in [18]). This hill-like function marks a dynamical stable profile of expression that in turn is defined as a 'coherent expression state (CES)' for a set of genes.

In the last work [18], we investigated the emergence of time-dependent formation of a CES on a space spanned by expression and temporal change in expression (which we call the *regulatory space*). The bifurcation of CES was observed in terms of the incremental change in a segment with a certain range of *nrmsf* (*v* < *nrmsf* < *v* + *r*: variable, *v* and a fixed value, *r*), which included the expression of thousands of mRNAs (refer to the bifurcation diagram of CES of the HRG response in Fig 5 in [18]). This bifurcation scenario revealed three distinct expression domains (refer to Table 1 in [18] with the relation: *rmsf = nrmsf*×*2*.*64*): dynamic domain: *nrmsf* > 0.16, transition domain: 0.08 <*nrmsf* < 0.16, and static domain: *nrmsf* < 0.08. Fig 1 shows the characteristic behavior of an expression profile going from unimodal to bimodal through flattening of the unimodal profile as the group average of *nrmsf* (<*nrmsf*>) decreases.

Interestingly, the smaller ensemble of mRNAs (*n* = 182) near CP also changes from a unimodal to a bimodal density profile, showing the existence of self-similar (unimodal-bimodal) power law behavior to that of the whole expression (Figs 1 and 3A). Therefore, we can safely affirm that the scaling behavior around the critical point together with its sandpile-avalanche type of singular behavior has the characteristics of *self-organized criticality* (SOC) [23–26].

Therefore, the evidence of SOC in the whole expression space through the unimodal-to-bimodal phase transition at CP, suggests that *nrmsf* plays a role analogous to the degree of fluctuation/freedom in statistical thermodynamics, where *nrmsf* is the order parameter discriminating three expression domains as distinct critical states in mRNA expression: super-, near- and sub-critical states. This behavior suggests the coexistence of three genomic multi-compartment structures:

1. Super-critical state: flexible genomic compartment corresponding to a dynamic domain (*N* = 3269 mRNA species) for a high variance of expression: *nrmsf* > 0.16 with a unimodal density profile. The most vivid early stress response in the super-critical state is revealed.
2. Near-critical state: equilibrated compartment corresponding to a transit domain (9707 mRNA species) for an intermediate variance of expression: 0.08 <*nrmsf* < 0.16 with a flattened unimodal profile. The critical point of the expression profile (*nrmsf*: 0.09) lies in the near-critical state at the boundary between low and high-expression, which suggests SOC-based phase transition occurs in the near-critical state.
3. Sub-critical state: rigid compartment corresponding to a static domain (9059 mRNA species) for a low variance of expression: *nrmsf*< 0.08 with a bimodal profile corresponding to high- and low-expression states. Genomic DNA phase transitions are expected to play an essential role in the regulation of low-variance gene expression (see the latter sections).

**Fig 4:**
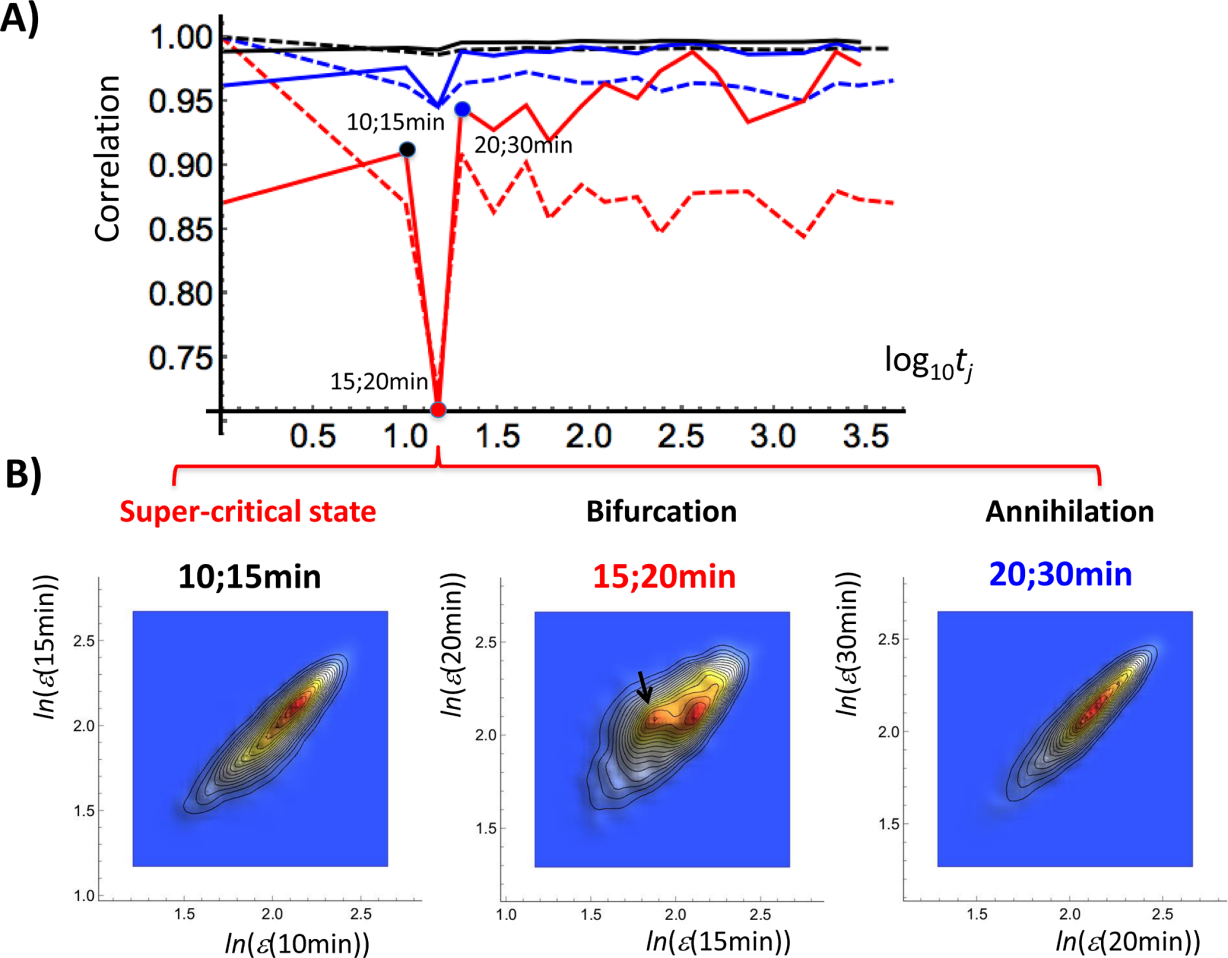
The early stage of a singular response. Panel A) shows the Pearson correlation (dashed line: *P*(*t*_0_;*t*_j_)) between the *t*_0_ expression profile and the expression profiles at increasing time. Solid line reports the correlation between neighboring temporal expression profiles (*P*(*t*_j_;*t*_j+1_)) relative to different characteristic domains (*x*: common logarithm of minutes; *y*: correlation value). Correlation dynamics reveal a sharp cleft at 15–30 min in the dynamic domain (super-critical: red) with less and slight effects on the transition (near-critical: blue) and static (sub-critical: black) domains, respectively. Panel B) shows that the singular response is due to the bifurcation of a coherent expression state (CES indicated by a black arrow, equivalent to HES2 in Fig 6A: right panel) at 15–20 min and its annihilation at 20–30 min (left: 10 min vs. 15 min; middle; 15 vs. 20 min; right; 20 min vs. 30 min in expression). This is related to the fast /short-span mode in SOC (see the main text).

Fig 4A shows the profile correlation dynamics for the HRG sub-critical (static), near-critical (transit) and super-critical (dynamic) ensembles, respectively. The y-axis shows the Pearson correlation coefficients with the initial (*t*_*0*_) condition along the entire mRNA expression profile for ensembles of three critical states. All three sets show a clear singularity (maximal displacement from the *t*_*0*_ profile) at 15–20 minutes that is much higher (as expected) for the dynamic domain (P(*t*_*0*_;*t*_*j*_) = 0.75). The dynamic domain shows a higher displacement from the initial condition with respect to the other domains across the entire time window (p< 0.0001, repeated-measures ANOVA).

### Genomic Avalanche: Onset of Scaling-Divergent Behavior at Critical Point

In our previous work [18,27–30], we observed, in distinct biological processes, the emergence of global asymptotic correlation trends. This was made possible by the grouping of mRNA expression by temporal change in expression and amount of temporal fluctuation.

To further investigate this phenomenon in the light of SOC, we performed correlation analyses of mRNA expression between *nrmsf* groups, while adopting different scaling options:

1. No scaling: the correlation is evaluated on the data as such;
2. Ensemble average of expression for each group at *t* = *t*_*j*_: i.e., the expression data are subtracted from the center of mass (CM_group_) of the group (Pearson correlation), and
3. Ensemble average of the overall expression: i.e., the expression data are subtracted from the center of mass of the entire genome at each time (CM_whole_) at *t* = *t*_*j*_.

**Fig 5:**
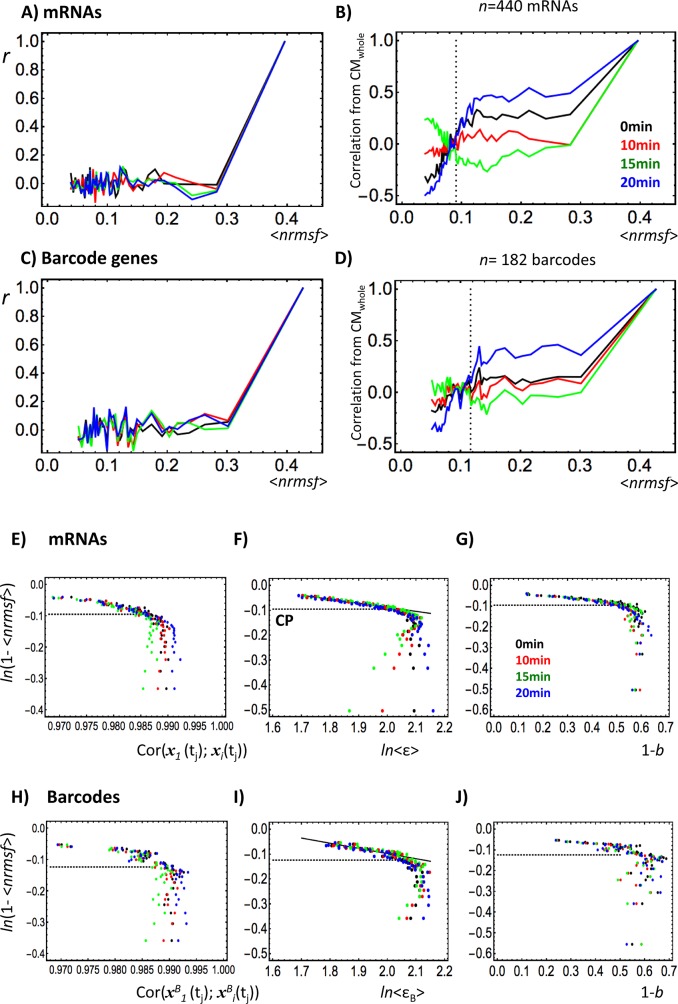
Emergent avalanche like distribution on between-profiles correlation analysis of expression groups. Whole mRNA expression (unit: mRNA) and barcode genes (unit: barcode gene; refer to the main text) are sorted and grouped (group size: 440 mRNAs; 182 barcodes) according to the degree of *nrmsf*. In A-D, the y-axis shows the degree of correlation of fluctuations from the initial point and the x-axis shows <*nrmsf*>: ensemble average of *nrmsf*. Correlations (0 min: black; 10 min: red; 15 min: green; 20 min: blue) were computed according to different scaling options (see the main text): A) Correlation scaled from the center of mass of the group (CM_group_) (Pearson correlation: *r*). Correlations fluctuate around zero, indicating that expression is stochastic in nature. B) Correlation from the center of mass of the whole expression (CM_whole_). A focal point (FP) is present, where the correlations converge at around the middle of the groups, and at this point their trends invert and start to diverge. This inversion reveals clear coupling with opposite coherent stochastic oscillations of the ensemble of expressions above and below the focal point (see the main text). Similar behavior is evident for barcode genes in C), in which correlations are scaled from CM_group_, and D) which in turn is scaled from CM_whole_. Dashed vertical lines in C) and D) show average *nrmsf* of CP (<*nrmsf*>_CP_) of mRNA and barcode genes over 10, 15 and 20 min, respectively are almost matched to *nrmsf* of FP, where average <*nrmsf*>_CP_ is 0.09 and 0.11 for mRNA and barcode gene, respectively. In E-G (first row: *N* = 22035 mRNAs), and H-J (second row: *N* = 7286 barcodes), the y-axis represents natural log of 1- <*nrmsf*>, *ln*(1- <*nrmsf*>). The x-axis represents left (E and H): the correlation with no scaling between groups at *t* = *t*_*j*_ between the highest *nrmsf* group (expression vectors: ***x***_***1***_ (*t*_j_), ***x*** ^B^_***1***_(*t*_j_) for mRNA and barcode gene, respectively) and the *i* ^*th*^ group (***x***_*i*_(*t*_j_) and ***x*** ^B^_*i*_(*t*_j_)); center (F and I): natural log of the average expression of a group (*ln*(<*ε*>) for mRNA and *ln*<*ε*_B_> for barcode gene, which show DEAB of the expression; right (G and J): the bimodality coefficient (1-*b*, and *b*: Sarle's bimodality coefficient) for mRNA expression and barcode genes, respectively. All figures for mRNA and barcodes show scaling-divergent behaviors, where CP shown by dashed horizontal lines (1- <*nrmsf*>_CP_) is at onset of the divergent point. This exactly reveals the characteristics of avalanche like distribution in the sandpile model of SOC. The power law scaling behaviors (F and I) on DEAB of the expression for mRNAs and barcode genes are revealed in the form of 1- <*nrmsf*> = < *ε* >^-*β*^: *α* = 1.27 & *β* = 0.16 (p< 10^−14^) and *α* = 1.37 & *β* = 0.21 (p< 10^−6^), respectively, which indicates similar SOCs of the mRNA (each gene contributes to the model) and barcode (the barcodes as single units) representations.

Pearson correlation clearly shows stochastic expression around group average (i.e., CM_group_): we observed near zero Pearson correlation (Fig 5A) between the highest *nrmsf* group and the *i* ^*th*^ group at *t* = *t*_*j*_, i.e., stochastic expression around CM_group._

This stochasticity tells us that correlation of expression between the highest *nrmsf* group and the *i* ^*th*^ group at *t* = *t*_*j*_ (Fig 5E) corresponds to the correlation of their CM_group_ (group averages) between expression and *nrmsf*, i.e., equivalent to the correlation behavior seen in *DEAB* (Dynamic Emergent Averaging Behavior) *of the expression* (Fig 5F). Therefore, we are seeing “between profile-correlation” in terms of correlation of the corresponding CM_group_ (see Materials and Methods).

Under the same heading, correlation computed with reference to CM_whole_ (Fig 5B) exhibits the profile-correlation of groups and amplifies the difference in correlation between different time points, which reveals the oscillatory behavior of whole-genome expression (see the next section). Notably, the correlation dynamics shows a clear focal point where different correlations converge at around the middle of the groups. Interestingly, CP corresponds both to the focal point (Fig 5B) in terms of *nrmsf* and to a point of divergent behavior in DEAB of the expression (Fig 5F).

As we pass through the critical (i.e., focal) point in DEABs of the expression, the trend of correlations in time inverts to show scaling-divergent behavior (Fig 5F), which suggests the CP is a point of the onset divergence, where order (scaling) and disorder (divergence) are balanced, i.e., the ‘genomic avalanche’ is shown at the critical point of SOC [31].

Regarding scaling behavior, the log-log plot of Fig 5F shows a power law behavior of ensemble average between variance of temporal fluctuation of expression (*<nrmsf>*) and mRNA expression (<*ε*>) described by 1-<*nrmsf*> = *α*<*ε*>^-*β*^ (*α* and *β* > 0)—higher <*nrmsf*> corresponding to higher <ε> in the sub-critical state (low variance expression). The power law relation suggests a quantitative relation between the physical plasticity of genomic DNA and gene expression regulation through DNA phase transition (refer to sub-critical barcode genes below). Natural logarithm of 1-<*nrmsf*> shows also the scaling-divergent behavior at CP in correlation of groups with no scaling and bimodality of groups (Fig 5E and 5G).

Sandpile-type singular behavior determines both the position of CP (Fig 2D) and the onset of scaling-divergent behavior (Fig 5F). The onset of scaling-divergent behavior happens at the CP, which stems from different mean fields: the position of CP from grouping based on expression change and the scaling-divergent behavior (i.e., DEABs) from grouping by *nrmsf*. This matching of critical behaviors across different mean fields provides further confirmation of the occurrence of SOC.

Therefore, the critical point of SOC coincides with focal point of global correlation behavior where the unimodal-bimodal transition through criticality occurs. The scaling-divergent profile in DEABs of the expression strongly resembles avalanche size distribution in the sandpile model [21], which sheds light on SOC in the global genetic response as a route for a spatio-temporal genomic phase transition.

### Coupling of Modes with Different Time-scales in Self-Organized Criticality

**Fig 6:**
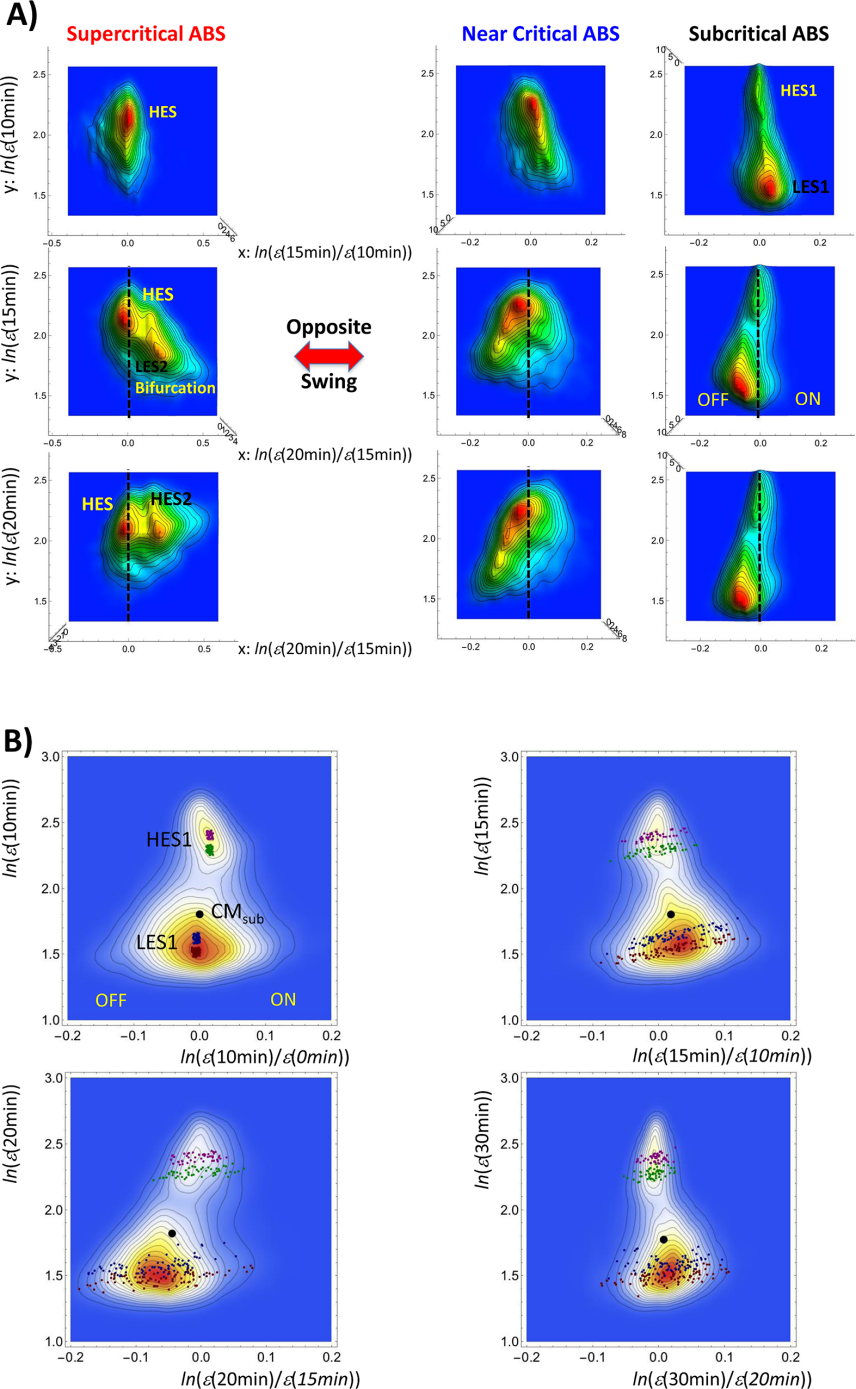
Anti-phase behavior of autonomous bistable switches (ABSs) between super- and sub-critical states. A) Pseudo-3-D probability density profiles on the regulatory space show the opposite ON-OFF oscillation of coherent expression states (low- and high-expression states (LES and HES) with *x* = 0 (marked by a vertical dashed line) in ABS between super- and sub-critical & near- critical states (left column: ABS of super-critical; center: near-critical: right: sub-critical). The *x*-axis shows the (natural log) expression change at 10–15 min (first row), 15–20 min (second), and 20–30 min (third); the *y*-axis shows the (natural log) expression for 10 min (first row), 15 min (second), and 20 min (third). In the super-critical ABS, LES2 is bifurcated at 15 min, becomes HES2 at 20 min, and annihilated at 30 min (refer to Fig 4B). B) Sensitivity to the initial conditions of the temporal dynamics of groups of mRNAs at different expression initially localized around *x* = 0 (indicated by purple, green, blue and red squares; left top) in a sub-critical ABS represented as a 2-D density profile (*N* = 9059 mRNAs). The groups refer to two coherent expression states–two groups are in a high-expression state (HES1), the other two in a low-expression state (LES1)—of the sub-critical ABS on the regulatory space (left top: 0–10 min vs. 10 min; right top: 10–15 min vs. 15 min; left bottom: 15–20 min vs. 20 min; right bottom: 20–30 min vs. 30 min). The figure reveals amplification in the expression change (*x*-axis) but not in expression (*y*-axis). This behavior indicates a highly correlative behavior for expression and stochastic resonance effect for the expression change. The black solid dot indicates the center of mass of sub-critical ABS(CM_sub_).

In this section, we address coherent stochastic oscillations (CSOs) in the time-dependent SOC and their coupling in critical states as to reveal the fast and slow modes of mRNA expression. The dynamical change reveals the formation of autonomous bistable switches (ABSs; Fig 6A) by a pair of coherent expression states (CESs) thus achieving a robust dynamic control of the genome-wide expression.

**Fig 7:**
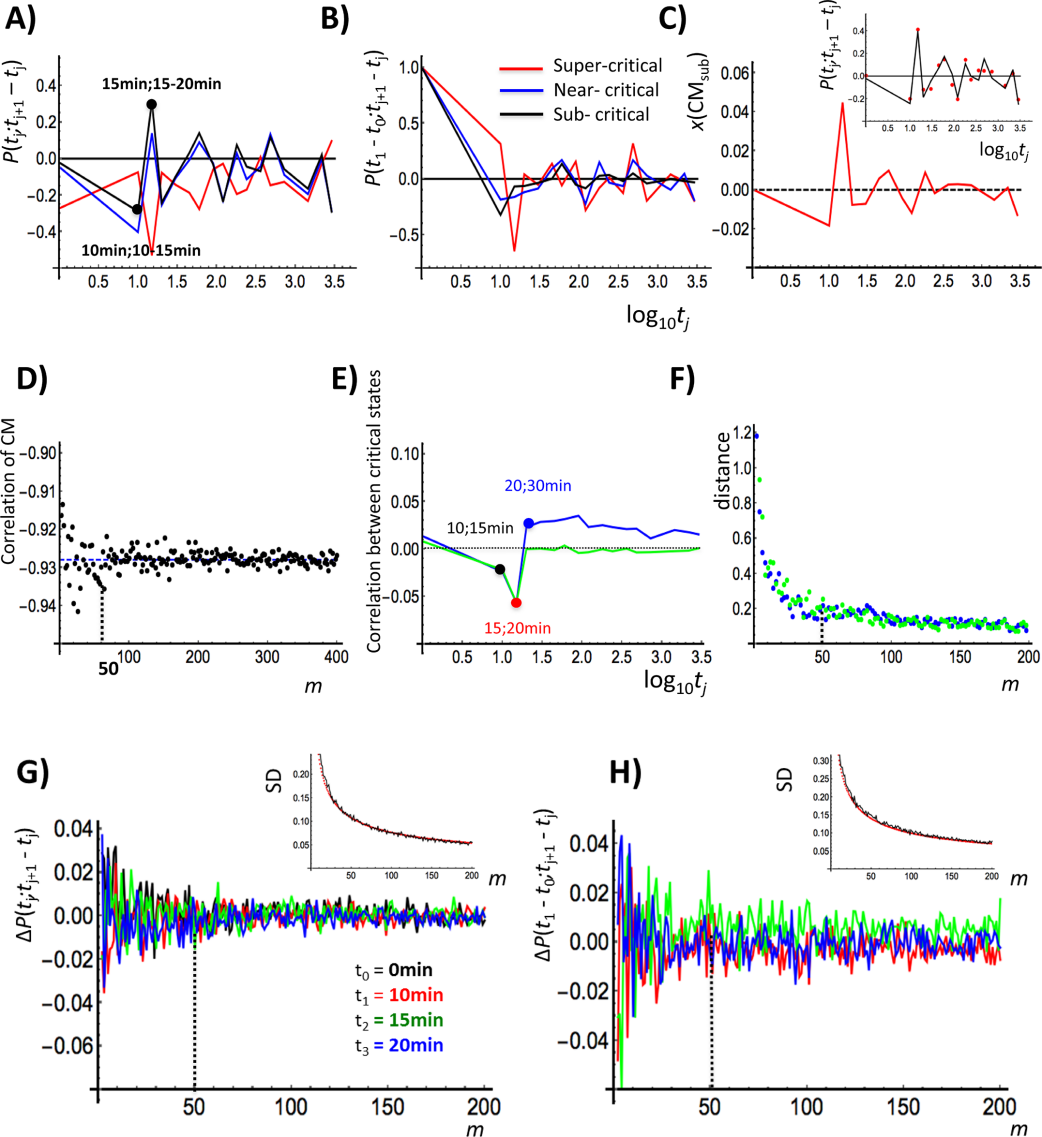
Coupling between fast and slow modes of coherent stochastic oscillation (CSO). CSO is appreciated in terms of Pearson correlations (*x*: common logarithm of minutes): A) between expression (at *t* = *t*_j_) and the expression change (change in expression from *t*_j_ to *t*_j+1_; *j =* 1,..,17), *P*(*t*_j_;*t*_j+1_−*t*_j_)) and B) in the difference in expression between 0–10 min and *t*_j+1_−*t*_j_, *P*(*t*_1_ *−t*_0_;*t*_j+1_−*t*_j_). In A), an opposite response is seen between the super-critical state (red line) and sub-critical (black) & near-critical (blue) states, which shows that the opposite coherent oscillatory dynamics of ABS continue, whereas B) shows the loss of the initial memory of the expression change (0–10 min), which confirms that the change in expression is stochastic. The x-axis represents log_10_(*t*_j_[min]). C): Temporal change in expression of the center of mass of ABS^sub^ (*x*(CM_sub_)) shows, albeit with slight oscillation around zero, a good correlation with the Pearson correlation, *P*(*t*_j_;*t*_j+1_ *- t*_j_) of ABS^sub^ (upper right; *x*(CM_sub_) by red dot), which reveals an algebraic correlation to the dynamics of CM_sub_ as a feature of SOC (see the main text for details), where the scaled motion of CM_sub_ (upper right) is multiplied by $\alpha \frac{N(t_{j})}{N(t_{j+1}-t_{j})}$, where *α* = 1.45 and *N*(*t*_*j*_) and *N*(*t*_*j*+1_ − *t*_*j*_) are normal to the expression vector at *t* = *t*_j_ and the vector of the expression change: *t*_j+1_ *−t*_j_. D): The long-span opposite dynamics shown in A) appear as an opposite sign of the Pearson correlation of CM between super- and sub-critical states. The average Pearson correlation (over 200 repeats; black dot) of the CM of a randomly selected temporal change in expression (*t*_j+1_−*t*_j_) from each critical state converges to *r* = -0.927 (*x*: *m* randomly selected mRNAs vs. *y*: Pearson correlation coefficient). E) Average Pearson correlation of expression (blue) and the change in expression (green) for random sampling (*m* = 100 with 200 repeats) between two critical states exhibits a similar singular fast/ short span correlation to the super-critical state at 15–30 min with no apparent subsequent response, which is confirmed by F): The figure reports converging Euclidean distance of two correlation points (*x*: time; y: correlation) between *m* and *m*+1 random samplings to zero as *m* is increased. This suggests the existence of coupling between a fast short span mode in the super-critical state and a long span coherent oscillation in the sub-critical state. G) and H): The emergence of coherent oscillation and stochasticity is examined in terms of G): the difference (Δ) of Pearson correlation, *P*(*t*_j_;*t*_j+1_−*t*_j_) between ABS^sub^(*t*_*j*_ *; t*_j+1_−*t*_*j*_) and *m* randomly selected mRNAs and H): the difference in *P*(*t*_1_ *−t*_0_;*t*_j+1_−*t*_j_) of *m* randomly selected mRNAs with 400 repeats for each choice, respectively (*t*_0_ = 0 min: black, *t*_1_ = 10 min: red, *t*_2_ = 15 min: green, *t*_3_ = 20 min: blue), where the standard deviation (SD) for time points (*j* = 1,2,3) follows $\alpha /\sqrt{m}$ scaling: α is 0.77 and 1.0 for coherence and stochasticity (in the inset figure in the upper-right corners; red: scaling; black: SD of *j* = 1), respectively. The results indicate the emergence of CSO after around *m* = 50, which is also supported by the random sampling results given in D) and F) (marked by black vertical dashed lines).

In critical states, two CESs form a pair to develop a pendulum oscillatory system, low-expression state (LES) swinging around high-expression state (HES), where in super-critical state, LES is bifurcated during the oscillation (Fig 6A; refer to Figs 7 and 9 in [18]). This pendulum-like oscillatory system acting as a pair of CESs is defined as an autonomous bistable switch (ABS). Furthermore, the oscillatory dynamics of ABS is shown to have a good correlation to dynamics of the center of mass of ABS (see the next section), which confirms an important characteristic of the profile-correlation (Materials and Methods).

Next, we depict how coherent stochastic oscillation (CSO) of ABS^sub^ occurs. Fig 6B shows the presence of coherent oscillation at an ensemble level and the stochasticity of a single gene expression (*Coherent Stochastic Oscillation*: CSO) in a sub-critical ABS. Coherent oscillation is defined as the oscillation on a density profile of ensemble of expressions, e.g., the oscillation of LES1 (containing thousands of mRNAs)—temporal ON-OFF swing (oscillation) of low-expression state (LES1) around high-expression (HES1) in the regulatory space. At the same time, stochastic expression within the sub-critical ABS is revealed in terms of sensitivity to the initial condition of mRNA expression groups. Fig 6B, shows, within sub-critical ABS, the sensitivity of mRNA expression dynamics to the initial conditions: over time, while the expression itself (y-axis) is little changed (i.e., nearly perfect correlation), its temporal swing (x-axis) is very protracted, amplified (i.e., correlation is nearly zero). Different non-overlapping initial groups within ABS^sub^ develop distinct collective motions. Initially localized expression groups spread over in expression change vs. expression space, while showing almost no apparent change as for expression. The high correlation as for expression is consistent with nearly perfect temporal Pearson correlation of the sub-critical state (Fig 4A).

The maximum change from the initial distributions happens in the expression (20 min) versus expression change (15–20 min) regulatory space, where singular like correlation response takes place in critical states, as clearly shown in Fig 4A. We observed that the maximum change in the expression width (*y*-axis) of groups occurs at 20 min for all groups while remaining almost invariant for other time points. On the other hand, notably, a large amplification—more than 10 times from the initial occurs in the expression change for all (four) groups. It is remarkable to see such an unexpected trend for the low-variance expression.

Therefore, with respect to CSO, where we observed the opposite behavior, here coherent expression stems from collective/coordinated fluctuation in the expression, whereas stochasticity with amplification occurs in fluctuation on the expression change. This amplification found even at the low-variance expression during the ON-OFF oscillation of LES1 is consistent with a stochastic resonance effect [32]. This clearly indicates the nonlinear nature of expression fluctuation dynamics.

We also observed CSO within super- and near-critical ABSs. This behavior exactly matches the macrostate/microstate opposition we sketched in the Materials and Methods. The above picture is confirmed by Pearson correlations, *P*(*t*_j_;*t*_j+1_−*t*_j_) and *P*(*t*_1_ *−t*_0_;*t*_j+1_−*t*_j_), which show an opposite correlation response of ABS between sub- and super-critical states in expression (*x*(*t*_j_) versus the change in expression: *x*(*t*_j+1_)–*x*(*t*_j_)) (Fig 7A), and stochasticity between changes in expression (*x*(*t*_1_)–*x*(*t*_0_) vs. *x*(*t*_j+1_)–*x*(*t*_j_)) (Fig 7B), respectively- confirming anti-phase dynamics of CSO in ABS between super- and sub-critical states on the regulatory space. Note: *x*(*t*_*j*_) = *ln*(*ε*(*t*_*j*_)) (natural log of mRNA expression at t = *t*_*j*_; symbolically represent by *ε*(*t*_*j*_)) and the change in expression: *x*(*t*_j+1_)−*x*(*t*_*j*_) = *ln*(*ε*(*t*_j+1_)/*ε*(*t*_*j*_)).

We represent super- and sub-critical ABS as ABS^super^(*t*_*j*_ *; t*_j+1_−*t*_*j*_) and ABS^sub^(*t*_*j*_ *; t*_j+1_−*t*_*j*_), respectively, in the regulatory space (Fig 6A).

To grasp the physical meaning of the opposite dynamics of two distinct CSOs, we examined the correlation of dynamics between ABS^super^(*t*_*j*_ *; t*_j+1_−*t*_*j*_) and ABS^sub^(*t*_*j*_ *; t*_j+1_−*t*_*j*_). In CSO, the coherent oscillation of ABS^sub^(*t*_*j*_ *; t*_j+1_−*t*_*j*_) is correlated with the dynamics of the center of mass (Fig 7C; see the next section), which reveals that the algebraic correlation of CSO is a feature of SOC [24]. Thus, the long-span opposite dynamics (seen until 72h) appears as an opposite sign of the Pearson correlation of the center of mass (*r* = -0.93) between two critical states (Fig 7D). Correlation of expression or of the change in expression between two critical states shows a similar singular behavior (Fig 7E) in the super-critical state at 15–30 min (Fig 4A), while thereafter no apparent response is observed. This suggests the existence of a fast short span mode in the super-critical state going together with long span coherent oscillation in the sub-critical state. This is again a signature of SOC, whose onset is marked by the coupling of fast/short- and slow/long-span modes [24].

The inversion of the correlation trend at the focal point (Fig 5B) reflects the coupling of two opposite oscillations of an ensemble of mRNA expression below and above the critical point (CP: *nrmsf* ~ 0.09) showing opposite temporal correlation trends (from 20min-0-10-15min to 15min-10-0-20min in the correlation order) for the above and below, respectively. Here the term ‘below’ (*nrmsf*< 0.08) indicates the sub-critical state and ‘above’ (*nrmsf*> 0.16) the super-critical state, passing through the near-critical state. The correlation response of the near-critical state (0.08 <*nrmsf*< 0.16) shows almost flat slope, which indicates constant correlation with CM_whole_ (see the next section). The presence of these distinct trends of correlation supports the existence of three critical states. The opposite response between super- and sub-critical states (Fig 6A) across FP is consistent with coupling of the fast and slow modes in mRNA expression dynamics as made evident by correlation and density analyses for characteristic critical states:

1. Fast / Short-span mode: this mode is evident in the super-critical state (dynamic domain, genes with a high temporal fluctuation of expression) in terms of a coherent expression state (CES) bifurcated at 15 min swinging around its partner. This bifurcation involves a change from a low-expression state (LES) to a high-expression state (HES) (Fig 6A; first column), which is annihilated at 20–30 min (Fig 4B). The response after 30 min becomes negligible (Fig 4A), which shows that the coherent expression state (CES) at 15–30 min in super-critical ABS is short-lived. This reveals that the CES is a metastable state and its dynamics reflects the transition of the metastable state. This shows the occurrence of a singular-like perturbation in genomic activity. This perturbation has a similar but smaller impact on the other critical states, which suggests that dynamic nonlinear interaction between critical states starts at the super-critical state.
2. Slow / Long-Span mode: this mode can be appreciated in the sub-critical state for low-variance mRNA expression. A pair of high- and low-expression states (HES and LES, respectively) exhibits sub-critical ABS (ABS^sub^(*t*_*j*_ *; t*_j+1_−*t*_*j*_)), showing ON-OFF swinging of LES around HES (Fig 6A and 6B). The profile-correlation P(*t*_*j*_;*t*_j+1_ *−t*_*j*_) in Fig 7A confirms the presence of a slow / long-span ON-OFF oscillation of ABS^sub^(*t*_*j*_ *; t*_j+1_−*t*_*j*_) that is correlated with the motion of its center of mass (Fig 7C).
3. Long-Span opposite dynamics of CSOs: ABS^sub^(*t*_*j*_ *; t*_j+1_−*t*_*j*_) exhibits opposite dynamics (i.e, anti-phase behavior) with respect to ABS^super^(*t*_*j*_ *; t*_j+1_−*t*_*j*_). As shown in Fig 7A, the profile-correlation P(*t*_*j*_;*t*_j+1_ *−t*_*j*_) shows opposite dynamics between sub- and super-critical ABSs, and near-critical ABS following oscillation similar to sub-critical ABS. This opposite dynamics continues until 72h, which reflects the existence of coupled CSOs between different critical states. This exhibits a long-span coordinating oscillatory activation (ON)-suppression (OFF) mechanism of gene expression dynamics. We previously reported that long-span dynamics were shared by different cell populations, from yeast to mammalian cells, in culture [33].

### Minimal Ensemble Size for Coherent Stochastic Behavior

Here, we address the threshold (in terms of the number of elements) at which coherent stochastic ensemble behavior emerges, and examine when randomly selected groups of mRNAs under an increasing number of genes start to display coherent stochastic behavior. We estimated this threshold in the sub-critical state by:

1. Invariance in terms of the Pearson correlation, P(*t*_j_;*t*_j+1_−*t*_j_) between ABS^sub^(*t*_*j*_ *; t*_*j+1*_−*t*_*j*_) computed on *k* randomly selected mRNAs with 400 repeats for each choice, and
2. Stochasticity in P(*t*_1_ *−t*_0_;*t*_j+1_−*t*_j_) of randomly selected mRNAs.

Under both approaches, the system converges after 50 randomly selected mRNAs (Fig 7G and 7H). The standard deviation for time points (*j* = 1,2,3) follows $\alpha /\sqrt{m}$ scaling (α is 0.77 and 1.0 for coherence and stochasticity, respectively). Random sampling analysis of the coupling of the two modes also supports the emergence of CSO after *m* = 50 (Fig 7D and 7F).

Importantly, coherent stochastic behavior is correlated with averaging behavior of the ensemble (i.e., the center of mass), so that emergent coherent behavior should satisfy a threshold condition for emergent mean field behavior following the law of large numbers (Fig 2B), where group size: *n* = 50 is the condition. This threshold also holds for super- and near-critical states; thus, as shown in Fig 2C, even a smaller ensemble of mRNAs from each critical state shows the scaling (unimodal-flattened-bimodal) to the whole expression (Fig 1). Interestingly, the same value of 50 genes as the threshold for the onset of coherent ensemble behavior was previously recognized in a completely different context and by different analytical techniques [28,29,34].

Therefore, we conclude that the minimal group size (*n*) of the emergent global response through SOC should be around *n* = 50, i.e., a coherent profile of an ensemble such as CSO is formed when its group size includes more than 50 elements.

### Algebraic correlation of the Coherent Stochastic Oscillation (CSO) of Sub-Critical Autonomous Bistable Switch (ABS)

Further we investigate whether the dynamical system of the sub-critical autonomous bistable switch, ABS^sub^(*t*_*j*_ *; t*_j+1_−*t*_*j*_)—ensemble of the low-variance of expression, leads to a characteristic algebraic correlation. We demonstrate that the coherent stochastic oscillation of sub-critical ABS^sub^(*t*_*j*_ *; t*_j+1_−*t*_*j*_) is coupled to the oscillation of its CM (Fig 7C), i.e., the algebraic correlation of ABS^sub^ to the dynamics of CM is characteristic of SOC.

The temporal Pearson correlation of the sub-critical state between expression and the change in expression, P(*t*_*j*_;*t*_j+1_ *–t*_*j*_) (Fig 7A) temporally oscillates between negative and positive correlations, which represents the ON-OFF oscillation of LES1 while HES1 does not show apparent oscillation in the sub-critical ABS (Fig 6B). This indicates that coherent oscillation of the ensemble stems from a good correlation to the oscillatory dynamics of its center of mass.

To elucidate the relationship of dynamics between ABS^sub^ and its CM, we examined the Pearson temporal correlation of fluctuating expression from its CM. The high correlation in temporal mRNA expression indicates

$${\text{ABS}}^{sub}\left(t_{j};t_{j+1}-t_{j}\right)\approx {\text{ABS}}^{sub}\left(t_{0};t_{j+1}-t_{j}\right).$$ (1)

Eq (1) further supports basic profile invariance (each tissue has a specific and robust expression profile) and thus explains why the Pearson correlation between expression at the initial (*t*_*0*_) and subsequent time points (*t*_*j*_; *j =* 1,..17), *P*(*t*_*0*_;*t*_*j*_) is nearly perfect (Fig 4A), whereas the Pearson correlation between the initial (*t*_*1*_ *−t*_*0*_) and subsequent differences in expression (*t*_j+1_−*t*_*j*_), *P*(*t*_1_ *−t*_0_; *t*_j+1_−*t*_j_) is near zero. Fig 7B shows how the memory of the change in expression at 0–10 min is lost due to its stochastic nature.

These results explain why the dynamics of ABS^sub^(*t*_*j*_ *; t*_*j+1*_−*t*_*j*_) is associated with a temporal Pearson correlation. For the pendulum oscillation of sub-critical ABS (Fig 6A; right panel), the Pearson correlation between the initial expression (*t*_*0*_) and the temporal expression change (*t*_*j+1*_−*t*_*j*_), *P*(*t*_*j*_; *t*_j+1_−*t*_*j*_), correlates with the oscillation of ABS^sub^(*t*_*j*_ *; t*_j+1_−*t*_*j*_) (Fig 7A). Interestingly, the temporal difference in CM in the mRNA expression, <*x*(*t*_*j*_)>–< *x*(*t*_j+1_)> is closely correlated with *P*(*t*_*j*_; *t*_j+1_−*t*_*j*_) (Fig 7C), i.e., the leading term of the geometric expansion of *P*(*t*_*j*_; *t*_j+1_−*t*_*j*_):

$$\begin{array}{l} P\left(t_{j};t_{j+1}-t_{j}\right)\approx P\left(t_{0};t_{j+1}-t_{j}\right)\\ \approx \alpha \cdot \lambda_{j}\left(\left\langle x\left(t_{j}\right)\right\rangle -\left\langle x\left(t_{j+1}\right)\right\rangle \right), \end{array}$$ (2)

where $\lambda_{j}=\frac{N\left(t_{j}\right)}{N\left(t_{j+1}-t_{j}\right)}$;

$$N\left(t_{j}\right)=\sqrt{\sum_{i=1}^{N}{\left(x_{i}(t_{j})-\left\langle x(t_{j})\right\rangle \right)}^{2}},\;N\left(t_{j+1}-t_{j}\right)=\sqrt{\sum_{i=1}^{N}{\left\{\left(x_{i}(t_{j+1})-\left\langle x(t_{j+1})\right\rangle \right)-\left(x_{i}(t_{j})-\left\langle x(t_{j})\right\rangle \right)\right\}}^{2}},$$

α is constant (~1.45), which is determined by the minimum Euclidean distance between *P*(*t*_*j*_; *t*_*j+1*_−*t*_*j*_) and approximate correlation (Eq (2)), and *P*(*t*_*j*_;*t*_*j*+1_ − *t*_*j*_) ≈ *P*(*t*_0_;*t*_*j*+1_ − *t*_*j*_) from Eq (1).

Eq (2) clearly reveals the oscillation of ABS^sub^(*t*_*j*_ *; t*_*j+1*_−*t*_*j*_) in terms of the CM of sub-critical ABS(*t*_j_ *; t*_j+1_−*t*_j_). With the use of Eq (4), the following relationship can be derived in terms of the leading term:

$$\gamma_{j}P\left(t_{0};t_{j+1}\right)-P\left(t_{0};t_{j}\right)\approx \alpha \cdot \left(\left\langle x\left(t_{j}\right)\right\rangle -\left\langle x\left(t_{j+1}\right)\right\rangle \right),$$ (3)

$$\left\langle x\left(t_{j}\right)\right\rangle -\left\langle x\left(t_{j+1}\right)\right\rangle \approx \frac{1}{\alpha }\left(\lambda_{j}-1\right),$$ (4)

where $\gamma_{j}=\frac{N\left(t_{j+1}\right)}{N\left(t_{j}\right)}$. With the use of Eq (3) with the approximation *P*(*t*_0_; *t*_*j*_) ≈ *P*(*t*_1_; *t*_*j*_) in the sub-critical ABS (refer to Fig 4A), a linear approximate term of *P*(*t*_1_ *−t*_0_; *t*_j+1_−*t*_j_) can be derived:

$$P\left(t_{1}-t_{0};t_{j+1}-t_{j}\right)\sim \beta_{j}\left(\left\langle x\left(t_{j}\right)\right\rangle -\left\langle x\left(t_{j+1}\right)\right\rangle \right)\;\text{for}\;j\geq 1,$$ (5)

where $\beta_{j}=\alpha \cdot \frac{\left(2\cdot N\left(t_{1}\right)-N\left(t_{0}\right)\right)}{N\left(t_{1}-t_{0}\right)N\left(t_{j+1}-t_{j}\right)}$.

Eqs (2) and (5) show that both Pearson correlations, *P*(*t*_*j*_; *t*_j+1_−*t*_*j*_) (Fig 7A) and *P*(*t*_1_ *−t*_0_; *t*_j+1_−*t*_j_) (Fig 7B), are correlated with the dynamics of CM of the sub-critical ABS(*t*_*j*_ *; t*_*j+1*_−*t*_*j*_), and the only difference is the coefficient. The linear term of *P*(*t*_1_ *−t*_0_; *t*_j+1_−*t*_j_) oscillates around near zero (ranged from -0.04 to 0.086), which captures the stochasticity in the change of expression, and *P*(*t*_*j*_; *t*_j+1_−*t*_*j*_) exhibits the temporal linear algebraic oscillation of the CM surrounding nonlinear stochastic expression dynamics, i.e., coherent stochastic oscillation of ABS^sub^(*t*_*j*_ *; t*_*j+1*_−*t*_*j*_). This algebraic correlation between ABS^sub^(*t*_*j*_ *; t*_*j+1*_−*t*_*j*_) and the CM confirms additional aspect on the characteristics of SOC. The result confirms the evidence of the profile-correlation even in the low-variance expression.

### Synchronization of Barcode Genes on Chromosomes with mRNA Coherent Expression Dynamics

The notion that coordinated genomic high-order DNA structural transitions underlie the observed gene expression dynamics requires further examination. In previous investigations we gave a proof-of-concept of the fact that the low-variance mRNA expression plays a crucial role in the global genetic response [8, 28]. This finding is at odds with the dominant picture of gene regulation that still focuses on ‘specific signature genes’ for each phenotype corresponding the most varying genetic elements. The importance of ‘low-variance’ genes points to genome acting as a whole (and as a ‘vehicle’ [8]) consistently with the material bases of genome organization composed of a two meters long molecule compressed into a few micrometers of nuclear space, therefore demanding for a coordinated folding/unfolding dynamics encompassing the whole genome.

The first step in the quest for gene expression observables that are suitable for establishing a link with transitional behaviors on chromosomes is the localization of the above-described gene expression domains on the chromosome.

In the preceding paragraphs we observed genome acting ‘as a whole’: the same transition point accounts for all the critical domains albeit with a different ‘amount of displacement’. If chromatin structural transitions are the material counterparts of such coherent dynamics, we can expect a corresponding typical arrangement of genes for critical states along the chromosomes.

**Fig 8:**
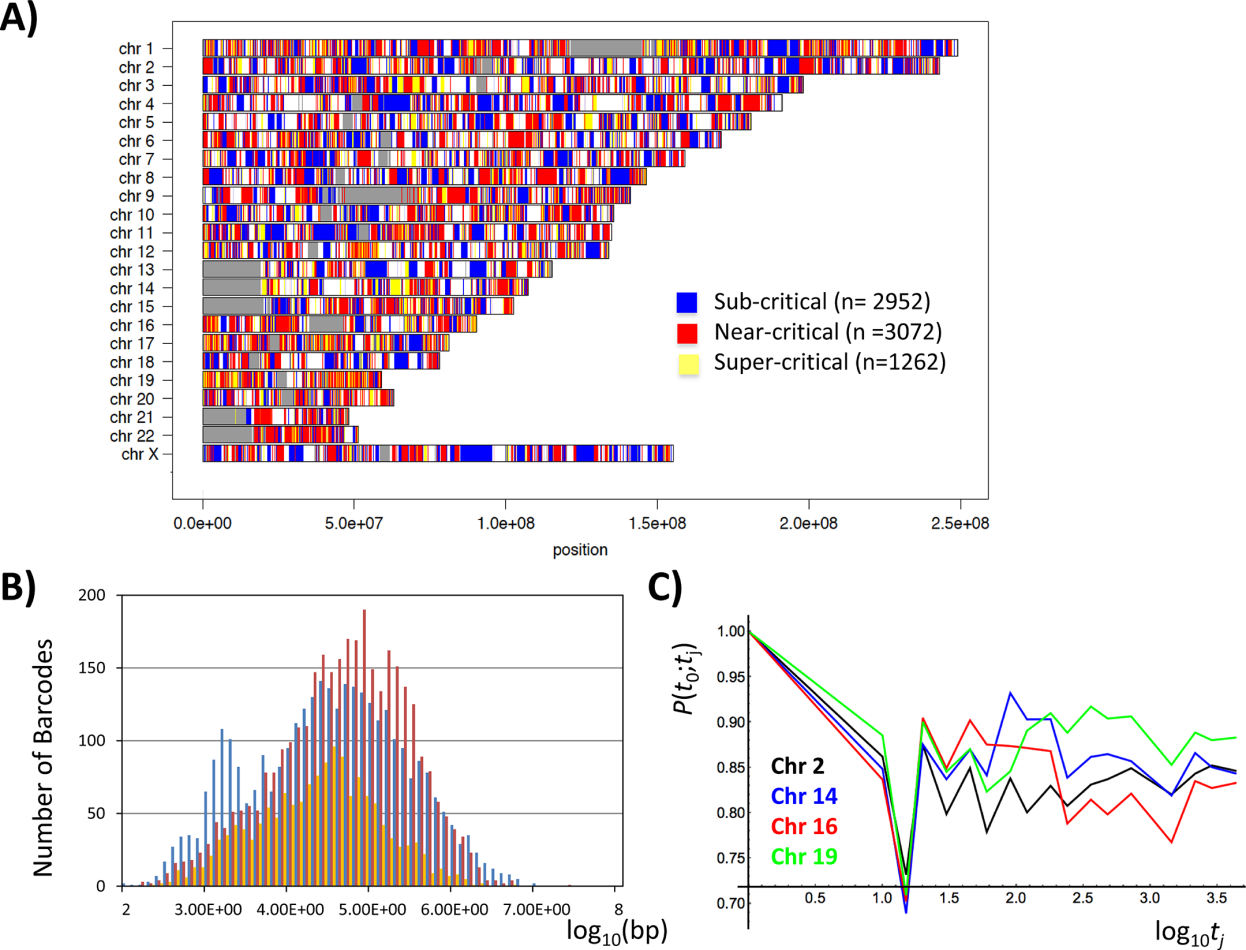
Barcode genes and temporal response of super-critical genes on chromosomes. A) Genes of critical phases are mapped into a human chromosome (UCSC hg19), corrected for the multiplicity of probes (mRNA expression) such as multiple probes of a gene due to an mRNA variant; the *x*- and *y*-axes show the chromosome position and chromosome number including X and Y chromosomes, respectively. Based on the distinct physical properties of the critical states, genes on a chromosome are clustered to form barcode genes (yellow: super-critical (*N* = 1262); red: near-critical (*N* = 3072); blue: sub-critical (*N* = 2952); gray: the unknown chromosome region), where the boundary of a barcode is defined as when two neighboring genes belong to different critical states; for instance, a sequence of.. S-S-S-T-T-S.. has two barcode genes, S-S-S and T-T; D: dynamic domain: super-critical; T: transit domain: near-critical; S: static domain: sub-critical (the plot was provided by I. E. Motoike). B) Frequency distribution of the correlation length of barcode genes for critical phases (the colors are the same as those in the barcode) shows the size of barcode genes within the range from kbp to Mbp for all states. The correlation length is estimated as the base length from the start codon of the initial gene to the end codon of the last gene within a barcode; the *x*- and *y*-axes show the common logarithm of a base pair and the number of barcodes (bin size: one-tenth of a unit length). C) Pearson correlation, *P*(*t*_0_;*t*_j_) of super-critical genes on chromosomes, which shows temporally four most responsive chromosomes (chromosome 2: black, 14: blue, 16: red, 19: green) with the singular like responses at 15–30 min (see also Fig 4A). The x-axis represents log_10_(*t*_j_[min]).

Three distinct states (super-, near- and sub-critical) in mRNA expression were revealed based on the theoretical framework of modern theoretical physics from a non-equilibrium self-organizing standpoint. We define consecutive genes pertaining to the same state as ‘barcode genes’ for each characteristic state on chromosomes (Fig 8A). Their size ranges from kbp to Mbp (7286 barcodes; Fig 8B), consistently with the characteristic scale of both the genomic swollen-compact transition [35] and the recently discovered topology-associated domains (TAD; see more in the Discussion) on chromosomes. Figs 2, 3 and 5 clearly show that gene expression on a group-basis exhibits self-organized criticality (SOC) in a manner very similar to that observed for the mRNA expression profile as such.

**Fig 9:**
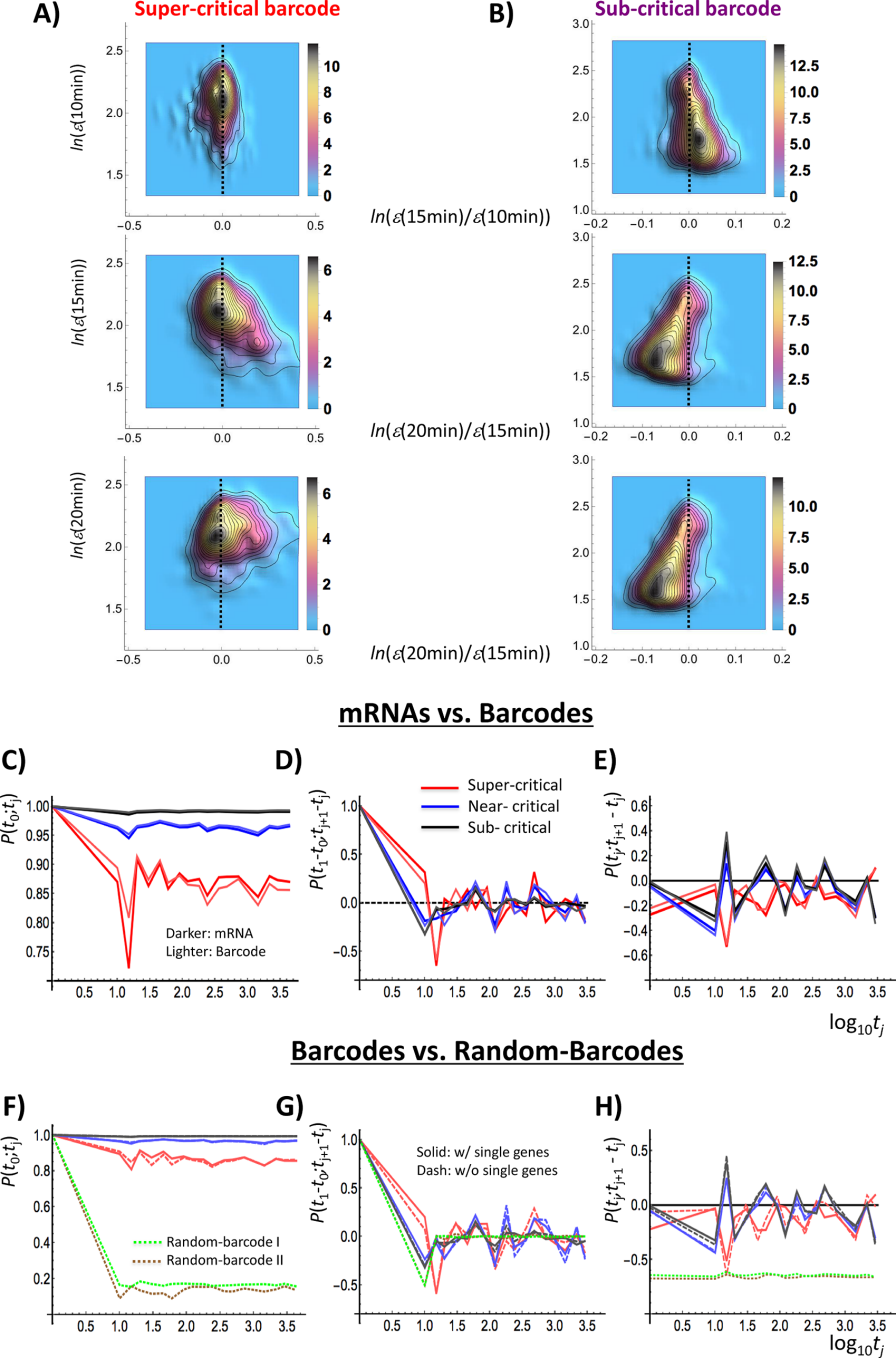
Synchronization of barcode genes on chromosomes with mRNA coherent expression dynamics. Panel A): super-critical state, Panel B): sub-critical state for barcode genes. The panels present the probability density functions of barcode genes on the regulatory space (first row: *x*: change in the natural logarithm of expression at 10–15 min; *y*: natural logarithm of expression at 10 min); second row: *x*: change at 15–20 min; *y*: at 15 min; third row: *x*: change at 15–20 min; *y*: at 20 min), showing a similar opposite oscillation between sub-critical and super-critical states in mRNA expression dynamics (Fig 6A). Panels C-E): mRNAs versus barcode genes. Panels F-H): a variety of barcode genes. Temporal Pearson correlations for sub-critical as well as near-critical barcode genes confirm the ON-OFF synchronization with mRNA expression dynamics, the same in terms of stochasticity and coherent oscillation (coherent stochastic oscillation: CSO; D: stochasticity; E: coherent oscillation), whereas super-critical barcodes reveal the opposite phase of CSO, showing similar temporal trend to critical states of mRNA (mRNAs (dark colors): subcritical: black; near-critical: blue; super-critical: red; barcodes: the corresponding lighter colors). Panels C & F: Pearson correlation, *P*(*t*_0_;*t*_j_); Panels D & G: *P*(*t*_1_-*t*_0_;*t*_j+1_ *-t*_j_), and Panels E & H: *P*(*t*_j_;*t*_j+1_−*t*_j_). Temporal Pearson correlations (F-H) confirm that barcodes without single gene (multiple genes: dashed lines) clearly follow similar trends of temporal correlations of the whole barcodes of critical states (solid lines), and thus similar correlation response to the mRNA dynamics. Correlation trends of barcodes are shown to be clearly distinct from random-barcodes: one (green dotted line: random-barcode I) for average value of randomly selected barcodes (*n* = 200) out of the whole barcodes combining critical states with 100 repeats–forming Gaussian distribution, and another (brown dotted line: random-barcode II) for barcode genes (*N* = 3130) of randomly mixed critical states, where genes on chromosome are randomly selected, and for each selected barcode, the number of elements is assigned randomly from 1 to 4 neighboring genes.

Next, we examined synchronized coherent stochastic oscillations (CSO) of barcodes to critical states of mRNA by checking if:

1. the observed synchronization is relative to single gene mRNAs, this will be the case if the majority of barcodes are made of a single gene, and if
2. the observed temporal correlations are distinct both from random sampling of barcodes and randomly mixed critical states in neighboring genes on chromosome (what we call ‘random barcodes I and II’, respectively; see more in Fig 9F–9H).

Regarding i), most of barcodes are single genes (86% in the super-critical; 53% in the near-critical; 58% in the sub-critical). This can be explained by the fact that in human genome, the mean size for protein-coding genes is about 27kbp [36] and that peaks of barcode size for critical state lie in the range of 30–60kbp (Fig 8B).

As demonstrated by the emergency of CSO with more than 50 elements, single gene barcodes guide CSO in SOC of barcodes; however, Fig 9F–9H reveals that barcodes containing multiple genes clearly follow similar trends of temporal correlations of mRNA of critical states. Furthermore, temporal correlations for coherent stochastic behavior of barcode genes are distinctively different from those of random barcodes. Most notably, randomly mixed critical states of genes (random barcodes II) on chromosomes do not display critical point (Fig 2D), which in turn confirms both the existence of critical states and the link between critical transition and the physical location on chromosomes. Hence, the results show that barcode genes are renormalized objects to synchronize coherent stochastic oscillation to mRNA expression dynamics of critical states, and are thus suitable units of genetic activity linked to coordinating transitional behaviors on chromosomes.

Notably, for the low-variance barcode genes in the sub-critical state, dominant expression regulation is expected by a genomic DNA swollen-compact transition led by interaction of genomic DNA with environmental molecules (i.e., association/dissociation of a large number of small molecules and ions with DNA under thermal fluctuation) [5,37]. Thus, CSO and ON-OFF synchronization of sub-critical barcode genes with sub-critical mRNAs (Figs 6A and 9B and 9E) suggests that barcode genes act in the interphase genome as the dynamical units of coherent ON-OFF phase transitions when chromosomes are in the sub-critical state.

## Discussion

### A Route for Genomic Phase Transition

**Fig 10:**
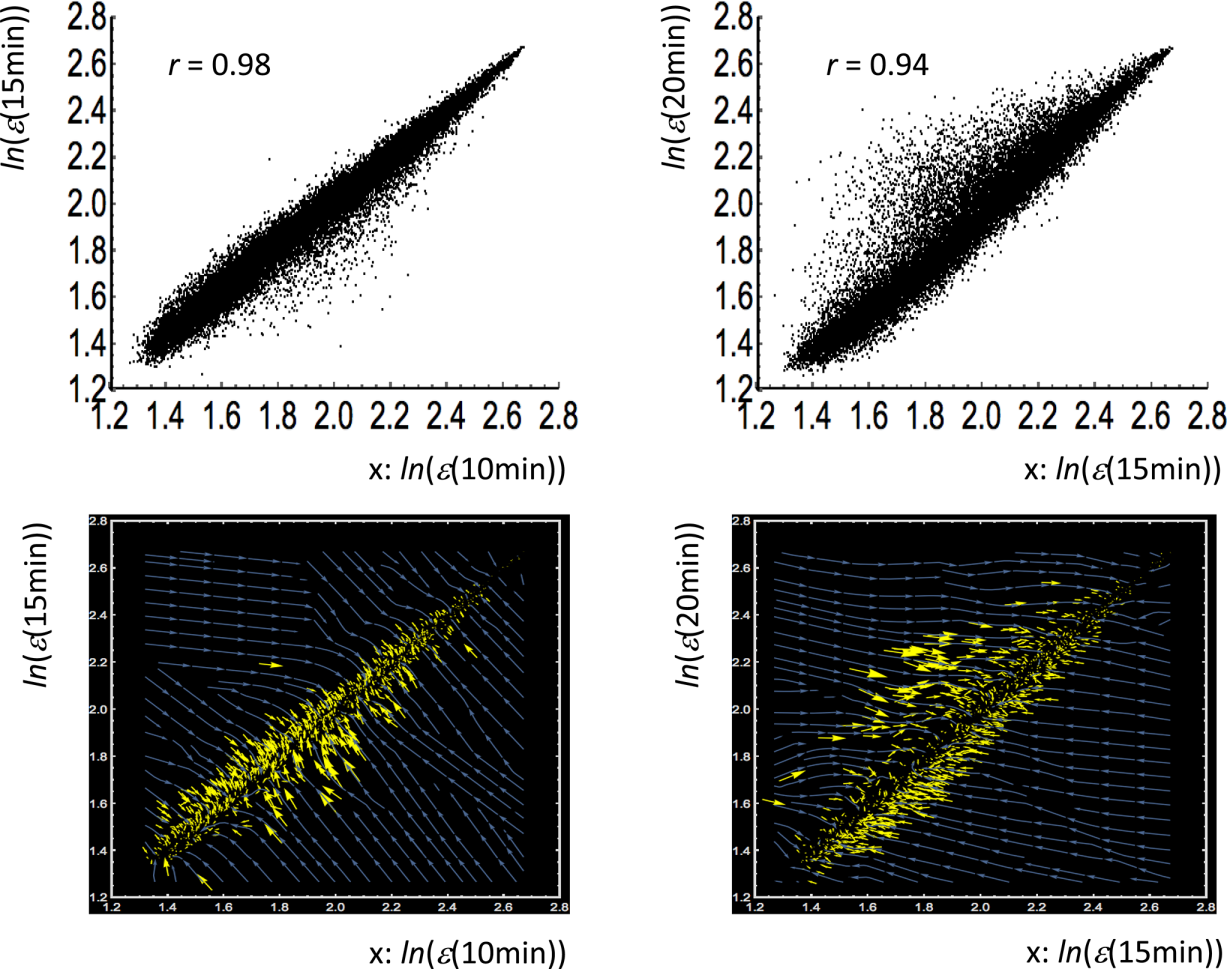
Genome-wide attractor on mRNA expression vector field. First row: profile-correlation of the whole expression between different time points (left: 10 min vs. 15 min; right: 15 min vs. 20 min) have a near to unity correlation—Pearson correlation coefficient, *r* = 0.98 in 10 min vs. 15 min (left), *r* = 0.94 in 15 min vs. 20 min (right). Each point represents single expression point, (*x*_*i*_(*t*_*j*_) = *ln*(*ε*(*t*_*j*_)), *y*_*i*_(*t*_*j*_) = *ln*(*ε*(*t*_j+1_)) (*i* = 1,..*N =* 22035), where *ln*(*ε*(*t*_*j*_)) is natural log of mRNA expression at *t* = *t*_*j*_ (*t*_*j*_ = 10 min, 15 min). The near to unity correlations between gene expression profiles coming from the same tissue implies that the global order of expression across different genes is largely invariant and is statistically very reliable. Second row: a stream plot (using Mathematica 10) is generated from vector field values {Δ*x*_*i*_(*t*_*j*_), Δ*y*_*i*_(*t*_*j*_)} given at specified expression points {*x*_*i*_(*t*_*j*_), *y*_*i*_(*t*_*j*_)}, where Δ*x*_*i*_ *= x*_*i*_(*t*_j+1_)- *x*_*i*_(*t*_*j*_), Δ*y*_*i*_ *= y*_*i*_(*t*_j+1_)- *y*_*i*_(*t*_*j*_). Streamlines of the whole expression vector field (blue lines) are generated and a yellow arrow represents a vector at a specified expression point (plotting every 20^th^ point). The result shows a clear “genome-wide attractor”—an attractor set is represented as a manifold (linear line: *y* = *rx*) in the space spanned by the whole mRNA expression of different time points, which suggests temporal invariance of whole-expression profiles acting as genome-wide attractors.

We demonstrated that the whole mRNA expression is self-organized through a critical state transition as a route for genomic phase transition to make the genome to act as an exquisite integrated expression system. Fig 10 reveals this genome integrated system as a “genome-wide attractor”—represented as a manifold (linear straight line) in the space spanned by the whole mRNA expression of different time points, which suggests temporal invariance of whole-expression profiles acting as the genome-wide attractors. It clearly shows the consilience between the statistical (near to unity Pearson correlation between gene expression profiles) and physical (field vectors) views of gene expression regulation.

Throughout our report, self-organized criticality (SOC) is shown to be the ‘driving force’ that attracts the global gene expression system toward a few preferred global states. Furthermore, the occurrence of SOC suggests the existence of a small number of control parameters for the genomic transition. The coherent synchronization of ON-OFF switching of the ensemble of barcode genes in a size range of kbp to Mbp as a scalable SOC of mRNA expression suggests that barcode genes are suitable observable units associated with the coordination of transitional behaviors at the chromosome level. This is consistent with the genomic DNA phase transition between compact and swollen conformations [37]. SOC of barcodes genes indicates coherent and fractal genomic DNA phase transitions.

Recently, due to advances in Hi-C methods, the role of topology-associated domains on a mega-base scale (TAD) in the regulation of the collective behavior of genes has begun to emerge. TADs represent physically separated clusters of co-expressed genes of sub-near-1Mbp length. A TAD structure is likely consistent with a fractal globule: a knot-free, polymer conformation that enables maximally dense packing while preserving the ability to easily fold and unfold any genomic locus [38–41].

Thus, a fractal globule structure of TAD could be the material counterpart at the basis of the observed coherent stochastic oscillations related to the sub-critical state in SOC of barcodes genes. The fact that such coherent behavior appears starting from a minimal number of 50 mRNA species (i.e., scaling behavior; Fig 7G and 7H) is a further, albeit indirect, proof of the above conjecture.

The elucidation of such a causal relationship might provide a dynamic picture of intra- and inter-chromosome interactions. The finding of fast/short-span (at 15–30 min) and slow/long-span (until 72h due to the experimental set-up [42]) oscillation modes and their coupling (Fig 7E) provides a possible scenario for linking via chromosome structural transition regulation and signal transduction. After the addition of HRG, which induces activation of the ErbB signaling pathway to generate an early stress response [42], a singular-like response occurs on the dynamic domain (super-critical state) at 15–30 min (Fig 4A) as the fast mode; interestingly most responsive genes at 15–30 min are from chromosomes 2, 14, 16 and 19 (Fig 8C **)**. This coupling suggests that through intra- and inter-chromosome interactions, the effect of the singular-like response spreads to the static domain (including the transit domain evident in Fig 4A), which exhibits ON-OFF coherent stochastic oscillation until 72h (slow mode).

The chromosome structural transition could be revealed by investigating the coherent phase transitions of specific regions of genomic DNA (e.g., TAD) to locate barcode genes for the fast mode. Coherent interaction with barcode genes of the slow mode could then in turn coordinate the initiation of coherent genomic DNA transitions, e.g., genome-wide chromatin state transitions [43], to provoke a global genetic response. Further studies are needed to assess the relative importance and mutual relation in cooperative behavior between complex key-lock epigenomic activities and genomic DNA transitions in this approach.

It is important that all possible scenarios consider the folding/unfolding transitions of chromatin, which is characterized by on/off switching of a DNA region on a scale of 10’s of kbp to Mbp.

### TAD Characterization of Barcode Genes

The DNA topology-associated domain (TAD) recently revealed by Hi-C techniques represents the single-cell- and tissue-invariant long-range unit of DNA loopy folding that approximates collectively transcribed linear gene arrays in sub-near-1 Mbp clusters [38–41]. The TAD structure was consistent with a fractal globule, a knot-free, polymer conformation that enables maximally dense packing while preserving the ability to easily fold and unfold any genomic locus. These gene clusters are physically separated by the insulator binding factor CTCF, and are enriched with architectural chromosome proteins [44], housekeeping genes, and SINE elements [45].

The fact that TAD clustered organization is driven solely by the chromosome structure in close proximity to the location of CTCF is of utmost importance. CTCF is an evolutionarily conserved zinc finger (ZF) protein that binds through the combinatorial use of target sites endowed with remarkable sequence variation. By analogy to the ‘hyper-variable’ portions of immunoglobulins, which allow for a rich repertoire of binding specificities, the formation of different CTCF–DNA complexes results in distinct regulations, including gene activation, repression and silencing [46]. All of these regulation patterns are closely associated with chromatin remodeling [47]. CTCF lies at the very center of epigenetic regulation linking differential gene expression and chromatin dynamics. The above results suggest that TADs are crucial operational units of the ‘genome field’ [48], i.e., the global dynamic organization of chromatin that supports its biological regulation.

Consecutive genes in the same phase (super-critical, near-critical and sub-critical) of the whole-genome transition are defined as individual ‘barcode gene groups’ of different chromosomes, have sizes within the range of TADs, from kbp to Mbp, and show the expected fractal structure. In turn, this size is consistent with the scale of the genomic loose (swollen) [35]—compact transition established earlier.

Based on their size, megadomains also correspond to the clusters of individual replicons or sub-chromosomal globules, which were visualized as foci in live cells by replication labeling with BrdU [49] or [50]. Clusters of replicons are supposed to be capable of cooperatively changing their conformation from compact to loose (swollen) due to DNA superhelicity of topologically constrained replicon-loops, which have mostly been studied in prokaryotes [51].

Taken together, these findings suggest that groups of barcode genes—50 barcodes for the minimum threshold of coherent stochastic oscillation—on a mega-base-sized scale are located along chromosomes. They are collectively transcribed and replicated by corresponding cell nucleus factories and can likely also undergo co-operative superhelicity transitions in a coherent stochastic manner. They represent the functional units of gene expression of self-organized criticality for physical phase transitions of the whole genome able to explain possible genome-wide chromatin state transitions [43]. The factors that are associated with the behavior of these units in the ‘genome field’ should be studied further.

### Genome as Neural Networks: Genome Computing

Finally, we address the potential implications of coherent genomic DNA transitions. The average expression of barcode genes in the static domain synchronizes with the ON-OFF oscillation of sub-critical autonomous bistable switch (ABS), which leads to a conjecture regarding the ON-OFF transition of the ensemble of barcode genes as coherent units of transitional behavior in genomic DNA and coherent genomic DNA transitions. Moreover, barcode genes on a chromosome show SOC very similar to that for mRNA expression. This indicates that barcode genes follow ABS dynamics similar to those of mRNA, such that the coordinated relationship between key-lock molecular transcriptional machinery and coherent genomic DNA on/off transitions in gene expression dynamics operate on the basis of characteristic critical states generated by SOC.

The time-dependent behavior of the genomic DNA transition follows a kinetic equation that shows cubic nonlinearity. This is due to a simultaneous change in the translational and conformational entropy of giant DNA together with surrounding counter ions to cause bimodality in the free energy, which in turn speeds the transition under cubic nonlinearity in the kinetic equation [52,53]. This cubic nonlinearity corresponds to the time derivative of the bimodal free energy functional [37,54]. Cubic-type nonlinearity plays an essential role in nervous system excitability; see, for example, the FitzHugh–Nagumo-type equation deduced from the Hodgkin-Huxley model, where the incorporation of negative feedback into cubic nonlinearity leads to the fundamental characteristics nerve firing [55,56].

Furthermore, self-organized criticality is observed in neuronal networks. Critical point of barcode genes shows the behavior of the sandpile model [20] (see Fig 2D) with an avalanche like distribution (Fig 5I), which is also shown in neural networks (neuronal avalanches: [31,57]).

Notably, this tells us that an ensemble behavior of barcode genes through on-off phase transitions on chromosomes has a similar dynamic ensemble behavior to a cascade of the on-off nerve firing bursts in neuronal networks. This indicates a non-trivial similarity between the coherent network of genomic DNA transitions and neural networks; coherent networks based on on/off switching of barcodes genes in SOC may imply the existence of rewritable self-organized memory in the genome acting as genome computing.

We suggest that computation by a self-organized network is an essential component of biological regulation, and in this study we assessed its relevance in the realm of genome dynamics and sketched some essential analogies with neural computation. Other phenomena, including protein folding and allosteric behavior, have also been studied in depth with respect to self-organized criticality (see, for example, [58]). In the case of the regulation of gene expression, which was the focus of the present work, we tried to fill the gap between very refined knowledge in terms of ‘key-and-lock’-type epigenetic mechanisms and the still largely unexplored ‘collective behavior’ of the genome as a whole.

## Conclusion

Global phase transition with a critical behavior in gene expression dynamics through a mean field approach was revealed in the early stress-like response to growth factors of the MCF-7 breast cancer cell line. This tells us that self-organized criticality (SOC) occurs as a form of genomic phase transition for dynamic control of the genome-wide gene expression. The cell population goes from a unimodal-bimodal transition in the gene expression distribution to the emergence of super-, near- and sub-critical states to reveal self-similarity of overall expression and a sandpile-avalanche type of singular behavior around the critical point (CP: *nrmsf* ~ 0.09). Spatial-temporal regulation of gene expression through SOC showed the generation of a fast and slow mode of (ON-OFF) oscillation coupled by autonomous bistable switches (ABSs) between sub- and super-critical states. Further deciphering molecular bases of the coupling of fast (within first 20 min) and slow (lasting for 72h) modes may shed more light on the role of induction of global genome-wide phase transition within cell nucleus (for establishment of cell differentiation).

Our finding of coherent stochastic oscillation (CSO) in ABS indicates that nonlinear interactions between fluctuations of gene expression stemming from single cell and interacting cell population such as intrinsic and extrinsic fluctuations [59] may disclose a biophysical mechanism of CSO in ABS of critical states. This emerging layer of a relevant collective regulation starting from a given minimal threshold number of elements (cells) should be related to stochastic resonance and allows to go beyond the automatic application of single cell models to interacting cell ensembles.

Chromosome mapping of genes relative to different critical states (domains) revealed fractal-like barcode gene entities in the size range of from kbp to Mbp. The mean field approach applied to barcode genes showed that they are almost identical to those of mRNA as well as ON-OFF synchronization in both the sub- and super-critical states of SOC.

These results implies that coherent chromatin phase transitions (collective behavior of ensemble of phase transitions in a coherent stochastic manner) between compact and swollen conformations corresponding to barcode genes constituting dynamical molecular system are in charge for the material basis of the expression regulation dynamics observed on mRNA. Furthermore, the genome avalanche with ensemble dynamics of barcode genes in a coherent stochastic manner suggests that genome can be considered as an autonomous computing device. Overall, the present study provides a novel viewpoint to sketch a biophysically realistic and biologically motivated model of genome-wide gene expression regulation that overcomes the lack of realism of a strict ‘lock-and-key’ approach.

## Materials and Methods

### Mean Field Approach: Grouping of mRNA expression and Dynamic Emergent Averaging Behaviors (DEABs)

We analyzed time-series Affymetrix GeneChip (Affymetrix U133A 2.0 chip) microarray data (Gene Expression Omnibus database ID: GSE13009; 22035 Affymetrix probe set IDs; 242 probes lacking chromosome information were excluded) relative to gene expressions in a MCF-7 breast cancer cell line under the addition of the ErbB receptor ligand HRG-b during the early stress response (experimental details in [42]). Expression data were normalized using the Robust Multichip Average (RMA) for further background adjustment and to reduce false positives [60–62]. Two replicas (rep 1 and rep 2) of the same experiment were taken into consideration. Rep 1 was analyzed in this report and rep 2 was used to reconfirm SOC in the whole mRNA expression.

The whole-genome mRNA expression (*N* = 22035) data set was sorted from the highest temporal fluctuation to the lowest, and the root mean square fluctuation (*rmsf*) of expression for each mRNA species was evaluated at 18 time points according to:

$$rmsf_{i}=\sqrt{\frac{1}{T+1}\sum_{j=0}^{T}{\left(\varepsilon_{i}(t_{j})-{\bar{\varepsilon }}_{i}\right)}^{2},}$$ (6)

where *rmsf*_*i*_ is *rmsf* of the *i* ^*th*^ mRNA, which has the expression, *ε*_*i*_(*t*_*j*_), at *t* = *t*_*j*_ (*j* = 0,..,17; *j* = 1,..,*N*); and ${\bar{\varepsilon }}_{i}$ is the average expression value of the *i* ^*th*^ mRNA over the 18 time points: *t*_0_ = 0, *t*_1_ = 10min, 15, 20, 30, 45, 60, 90min, 2h, 3, 4, 6, 8, 12, 24, 36, 48, *t*_T = 17_ = 72h.

To reveal emergent properties of self-organized criticality, we define a normalized *rmsf as nrmsf*, where *nrmsf*_*i*_ ranges from 0 to 1 by dividing *rmsf*_*i*_ by the maximum of overall *rmsf* (i.e., 2.64 in Rep 1). We consider that the choice of *nrmsf* for ordering mRNA expression stems from the physical entity of gene expression scaling with the fractal aggregation state of chromatin. Here, we need to note that we may have another choice of normalized *rmsf* such as coefficient of variation (CV)–*rmsf*_*i*_ divided by temporal average expression of each mRNA, ${\bar{\varepsilon }}_{i}$; however, CV is not a good variable for analysis of expression phase transition to represent a degree of fluctuation since it explicitly eliminates gene expression level from entity of temporal variation, which means the choice of CV can loose a physical relationship between mRNA expression and fractal aggregation state of the chromatin.

It is important to stress that a low (high) *nrmsf* value does not imply that the corresponding expression is low (high), but only refers to the relative amplitude of temporal fluctuations. Next, we divided the sorted genes into *k* groups with an equal number *n* of mRNAs in the genome (*k* = *N/n*), where *k* is an integer of *N/n*; *n* is the number of mRNAs. Ensemble averages <*nrmsf*> and <*ε*> are defined as the simple arithmetic mean over an ensemble or a group of mRNAs.

As the group size *n* (number of probes in each group) increases, a nonlinear correlation (*dynamic emergent averaging behavior*: DEAB) emerges between the logarithm of mRNA expression and temporal variability (*ln*<(*ε*(*t*_*i*_)> versus *ln*(1- <*nrmsf*>)), where the brackets < > denote the ensemble/group average, and *ε*(*t*_*i*_) reflects mRNA expression at time *t*_*i*_ (*i =* 0,1,..,17). Note: originally DEAB of the expression was presented in the space of <*rmsf*> versus <*ln*(*ε*(*t*_*i*_))> (refer to Fig 1 in [18]).

DEAB of the expression at time *t* revealed a nonlinear correlation between the average values of expression and *nrmsf* at a fixed time point. When we compare the DEAB of the expression between different time points, coordinated motion of the ensemble of mRNA expression emerges according to the degree of the temporal fluctuation of mRNA expression (i.e., *nrmsf*). Most interestingly, overlap of different time points of DEAB of the expression reveals scaling-divergent behavior, where a point of onset of divergent behavior is critical point of SOC, consistent with avalanche size distribution of SOC. The response to HRG was much more marked than that to EGF, and thus we concentrated on the HRG data.

### Estimating correlations

The entire work builds upon the estimation of the temporal course of gene expression correlations as observed at different scales and by different measurement paradigms. Thus, it is of utmost importance to clarify the nature of the studied correlations. The most important point is to keep the between-profiles correlation separate from the between-genes correlation. These two are conceptually different views of the same ensemble of data and correspond to the analysis of R- and C- transposed spaces [63].

As reported by Giuliani [64], Between-Profiles correlations (C-space: samples as variables, columns / genes as statistical units, rows) rely upon the presence of a characteristic tissue-specific expression value (i.e., center of mass in expression: CM) for each gene. The entire range of expression CMs across the genome spans four orders of magnitude, while the variation for each single gene expression is considerably lower (see, for example, [64]). This situation naturally leads to close-to-unity positive correlations between independent samples relative to the same kind of tissue for the C-space.

On the contrary, Between-Genes correlations (R-space: samples as statistical units, rows / genes as variables, columns) are based upon the relatively small and noisy within-gene fluctuations and thus are considerably lower (Average Pearson *r* around 0.15–0.20) than Between-Profiles correlations, and have both positive and negative values.

Notably, between-genes correlations can be biased by spatial noise inside the same chip [65], while between-profiles correlations, which are based on comparisons of different chips, does not suffer this bias.

The fluctuation of the expression of a single gene around its ‘tissue-specific’ average allows for adaptation of the cell population to different environmental cues, while maintaining a substantially invariant global profile. In statistical mechanics terms, we can consider the largely invariant, tissue specific, genome-wide expression profile (C-space) as the macrostate (refer to genome-wide attractor in Fig 10), while the between-genes varying mutual correlations are the corresponding microstates. Moreover, the whole expression profiles are the proper points of the multidimensional phase space of genome regulation.

In our work we strictly refer to between-profiles correlations that were computed not only for gene expression values, but also for several observables that are associated with gene fluctuations over time. While between-profiles correlations for expression values are bound to be very high and positive due to the existence of a largely invariant CM profile, the between-profile correlations of the temporal variation of expression can show very different values following the dynamics of gene expression regulation. It is important to stress that between-profiles correlations for the change in expression can be stochastic, and moreover, stochastic resonance is expected to reveal in terms of nonlinear nature of such stochastic fluctuation, e.g., in coherent stochastic oscillation (CSO) of critical states of expression throughout our work.

The correlation estimation follows from expansion of the usual Pearson product moment correlation coefficient according to the formula:

$$\mathrm{cor}(x_{1}\left(t_{j}\right)-r_{1};x_{k}\left(t_{j}\right)-r_{k})=\frac{\sum_{i=1}^{n}\left(x_{{}_{1}}^{i}\left(t_{j}\right)-\left\langle r_{1}^{i}\left(t_{j}\right)\right\rangle \right)\left(x_{{}_{k}}^{i}\left(t_{j}\right)-\left\langle r_{{}_{k}}^{i}\left(t_{j}\right)\right\rangle \right)}{\sqrt{\sum_{i=1}^{n}{\left(x_{{}_{1}}^{i}\left(t_{j}\right)-\left\langle r_{1}^{i}\left(t_{j}\right)\right\rangle \right)}^{2}\sum_{i=1}^{n}{\left(x_{{}_{k}}^{i}\left(t_{j}\right)-\left\langle r_{{}_{k}}^{i}\left(t_{j}\right)\right\rangle \right)}^{2}}},$$ (7)

In (1), vector ***x***_1_(*t*_j_) is the expression vector of the highest *nrmsf* group. Vector ***x***_*k*_(*t*_j_) is the expression vector for the *k* ^th^ group, and the reference vector ***r***_*k*_ is taken to be either the zero vector (no reference scaling) or the center of mass vector in the *k* ^th^ group expression or the center of mass vector in the whole expression at *t* = *t*_*j*_ according to the different scaling paradigms adopted.

The different scaling options used in this work include:

1. The center of mass vector for the *k* ^th^ group expression at *t* = *t*_*j*_: $r_{k}=\sum_{i=1}^{n}\frac{\ln\left(\varepsilon_{{}_{i}}^{k}\left(t_{j}\right)\right)}{n}\left(1,1,..,1\right)$ (*n* = elements in the group) with the *i* ^th^ mRNA expression, $\ln\left(\varepsilon_{i}^{k}\left(t_{j}\right)\right)$ in the *k* ^th^ group, or
2. The center of mass vector for the whole expression at *t* = *t*_*j*_: $r=r_{k}=\sum_{i=1}^{N}\frac{\ln\left(\varepsilon_{i}\left(t_{j}\right)\right)}{N}\left(1,1,..,1\right)$ (*N* elements; *N* = 22035, total number of genes).

### A systemic mean field procedure to find a critical point of SOC

A systemic mean field procedure is developed to estimate and confirm a critical point (CP) of SOC of expression, which consists of the following steps:

1. Finding of CP: investigate whether a sandpile type of transition occurs: sort and group the whole mRNAs or barcode genes (*n* elements: *n*> 50) according to the degree of temporal change in expression (*t*_j+1_−*t*_*j*_) (*j* = 0, 1, 2,..) to find a singular (sandpile type) point as a critical point of SOC in space of both expression change vs. expression, where average *nrmsf* and expression values of CP over time points, <*nrmsf*>_CP_ and *ln*<*ε*(CP)>, respectively are determined,
2. Grouping of whole expression by *nrmsf*: sort and group the whole mRNAs or barcode genes (*n* elements) according to the degree of *nrmsf*,
3. Confirmation of CP based on the behavior of avalanche like distribution: generate temporal DEABs of the expression from group coordinate values {*ln*<*ε*(*t*_*j*_)>, *ln*(1-<*nrmsf*>)} (*j* = 0,1,2,..) to show a scaling-divergent behavior, where CP is located at the onset of it, and
4. Confirmation of CP based on self-similar power law behavior: plot the 3D density profile of an ensemble of expression to confirm the existence of self-similar power law behavior around CP to that of the whole expression, which is one of the characteristics of SOC.
